# Supplementary material for: Prevalence and factors associated with Tungiasis among school age children in Sub Saharan Africa: A systematic review and meta-analysis
Source: PLoS One. 2025 May 8;20(5):e0321513. doi: 10.1371/journal.pone.0321513 (PMC12061120; doi:10.1371/journal.pone.0321513)
Supplement: S2 File — (DOCX) [file pone.0321513.s002.docx]

**Results of JBI Quality Assessment**

| Studies | Clear eligibility criteria | Description of study subject and study setting | Valid and reliable method to measure the exposure | Standard criteria used for measurement of the condition | Identification of confounding factors | Develop of strategies to deal with confounding factors | Valid and reliable method to measured outcomes | Appropriate statistical analysis | Total score out of 8 | Quality  of score (100%) | Level of risk |
| --- | --- | --- | --- | --- | --- | --- | --- | --- | --- | --- | --- |
| Mtunguja (2023) | Yes | Yes | Yes | Yes | Yes | Yes | Yes | Yes | 8 | 100 | low |
| Mwai(2020) | Yes | No | Yes | Yes | No | No | Yes | Yes | 5 | 62.5 | moderate |
| Mwai(2022) | Yes | Yes | Yes | Yes | Yes | Yes | Yes | Yes | 8 | 100 | low |
| Mba(2022) | Yes | Yes | Yes | Yes | Yes | Yes | Yes | Yes | 8 | 100 | low |
| Amare (2021) | Yes | Yes | Yes | Yes | Yes | Yes | Yes | Yes | 8 | 100 | low |
| Tamene(2021) | No | Yes | Yes | Yes | Yes | Yes | Yes | Yes | 7 | 87.5 | low |
| Jorga(2020) | No | Yes | Yes | Yes | Yes | Yes | Yes | Yes | 7 | 87.5 | low |
| Joseph(2010) | Yes | Yes | Yes | Yes | No | No | Yes | Yes | 6 | 75 | low |
| Walker(2017) | No | Yes | Yes | No | No | No | Yes | Yes | 4 | 50 | moderate |
| Girma(2018) | No | Yes | Yes | Yes | Yes | Yes | Yes | Yes | 7 | 87.5 | low |
| Nsanzimana(2019) | No | Yes | Yes | Yes | Yes | Yes | Yes | Yes | 7 | 87.5 | low |
| Ugbomoiko(2016) | No | Yes | Yes | Yes | No | No | Yes | Yes | 5 | 62.5 | moderate |
| Samuel (2017) | No | Yes | Yes | Yes | No | No | Yes | Yes | 5 | 62.5 | Moderate |
| Ngunjiri(2015) | Yes | Yes | Yes | Yes | No | No | Yes | Yes | 6 | 75 | low |
| Mwangi(2015) | Yes | Yes | Yes | Yes | Yes | Yes | Yes | Yes | 8 | 100 | low |
| Ugbomoiko (2007) | No | Yes | Yes | Yes | No | No | Yes | Yes | 5 | 62.5 | Moderate |
| Mazigo(2012) | No | Yes | Yes | Yes | No | No | Yes | Yes | 5 | 62.5 | moderate |
| Anyaele(2021) | No | Yes | Yes | Yes | No | No | Yes | Yes | 5 | 62.5 | Moderate |
| Wiese(2017) | No | Yes | Yes | Yes | Yes | Yes | Yes | Yes | 7 | 87.5 | Low |
| Okoth(2015) | Yes | Yes | Yes | Yes | No | No | Yes | Yes | 6 | 75 | Low |
| Albert(2016) | No | Yes | Yes | Yes | No | No | Yes | Yes | 5 | 62.5 | Moderate |
| Collins(2009) | No | Yes | Yes | No | No | No | Yes | Yes | 4 | 50 | Moderate |
| Ndung’u(2015) | Yes | Yes | Yes | No | No | No | Yes | Yes | 5 | 62.5 | Moderate |
