# Supplementary material for: Prevalence and factors associated with Tungiasis among school age children in Sub Saharan Africa: A systematic review and meta-analysis
Source: PLoS One. 2025 May 8;20(5):e0321513. doi: 10.1371/journal.pone.0321513 (PMC12061120; doi:10.1371/journal.pone.0321513)
Supplement: S4 File — (DOCX) [file pone.0321513.s004.docx]

Extracted literatures

| **S.no** | **Title** | **Name of data extractors** | **Date of data extraction** | **Reason for exclusion** |
| --- | --- | --- | --- | --- |
|  | A Syndromic Approach for Diagnosing and Managing Travel-Related Infectious Diseases in Children | Gete B. & Leykun B. | March 7,2024 | Title & abstract |
|  | A bird-parasitic fly, Carnus hemapterus Nitzsch (Diptera, Carnidae) in Japan: avian hosts, infestations, and a case of human dermatitis caused by adult | Gete B. & Leykun B. | March 18, 2024 | Title & abstract |
|  | A Brazilian child with a nodule in the foot | Gete B. & Leykun B. | March 18, 2024 | Title & abstract |
|  | A case of atopic dermatitis caused by Ascaris lumbricoides infection | Gete B. & Leykun B. | March 18, 2024 | Title & abstract |
|  | A case of imported Tungiasis | Gete B. & Leykun B. | March 18, 2024 | Title & abstract |
|  | A case of imported tungiasis in Scotland initially mimicking verrucae vulgaris. | Gete B. & Leykun B. | March 18, 2024 | Title & abstract |
|  | A Case of Non-cutaneous Kaposi Sarcoma | Gete B. & Leykun B. | March 18, 2024 | Title & abstract |
|  | A case of severe tungiasis in Nigeria | Gete B. & Leykun B. | March 18, 2024 | Title & abstract |
|  | A case of tularemia presenting as a dental abscess: case report | Gete B. & Leykun B. | March 18, 2024 | Title & abstract |
|  | A case of tungiasis | Gete B. & Leykun B. | March 18, 2024 | Title & abstract |
|  | A Case of Tungiasis after Traveling to Congo | Gete B. & Leykun B. | March 18, 2024 | Title & abstract |
|  | A case of tungiasis in Israel | Gete B. & Leykun B. | March 18, 2024 | Title & abstract |
|  | A case report of Sarcoptes scabiei infection in Ahwaz, Iran | Gete B. & Leykun B. | March 18, 2024 | Title & abstract |
|  | A Clinical Study of the Pattern of Dermatoses among Schoolgoing Children Attending a Tertiary Care Center in North Karnataka | Gete B. & Leykun B. | March 18, 2024 | Title & abstract |
|  | A comparative study of impairment of quality of life in children with skin disease and children with other chronic childhood diseases | Gete B. & Leykun B. | April 3, 2024 | Title & abstract |
|  | A comparison of clinical, radiological, and quality-of-life outcomes of double-plate internal and Ilizarov external fixations for Schatzker type 5 and 6 tibia plateau fractures | Gete B. & Leykun B. | April 3, 2024 | Title & abstract |
|  | A cross-sectional study of dermatological problems among differently-abled children | Gete B. & Leykun B. | April 3, 2024 | Title & abstract |
|  | A hemorrhagic-crusted papule with a central crater on the heel. Tungiasis | Gete B. & Leykun B. | April 3, 2024 | Title & abstract |
|  | A jigger of alcohol, a dash of depression, and bitters: a suicidal mix | Gete B. & Leykun B. | April 3, 2024 | Title & abstract |
|  | A mammary myiasis caused by Psychoda albipennis (Diptera: Psychodidae) in a dairy cow: first record | Gete B. & Leykun B. | April 3, 2024 | Title & abstract |
|  | A Mathematical Model of Thermography with Application to Tungiasis Inflammation of the Skin | Gete B. & Leykun B. | April 3, 2024 | Title & abstract |
|  | A mixed-methods approach to understanding domestic dog health and disease transmission risk in an indigenous reserve in Guyana, South America | Gete B. & Leykun B. | April 3, 2024 | Title & abstract |
|  | A Neglected Public Health Problem in Rural Cameroon | Gete B, Leykun B., Belay D. | April 3, 2024 | Included |
|  | A new infusorian parasite in sand fleas | Gete B. & Leykun B. | April 3, 2024 | Title & abstract |
|  | A new species of Tunga (Siphonaptera) from Ecuador | Gete B. & Leykun B. | April 3, 2024 | Title & abstract |
|  | A new species of Tunga (Siphonaptera: Tungidae) from Brazil with a key to the adult species and neosomes | Gete B. & Leykun B. | April 3, 2024 | Title & abstract |
|  | A new species of Tunga (Siphonaptera: Tungidae) parasitizing cattle from Brazil | Gete B. & Leykun B. | April 3, 2024 | Title & abstract |
|  | A new species of Tunga perforating the osteoderms of its armadillo host in Argentina and redescription of the male of Tunga terasma | Gete B. & Leykun B. | April 3, 2024 | Title & abstract |
|  | A new Tunga (siphonaptera) from the nearctic region with description of all stages | Gete B. & Leykun B. | April 3, 2024 | Title & abstract |
|  | A painful, draining black lesion on the right heel. Tungiasis | Gete B. & Leykun B. | April 3, 2024 | Title & abstract |
|  | A patient returning from Africa finds a mass imbedded in the skin of her right foot. Tungiasis | Gete B. & Leykun B. | April 3, 2024 | Title & abstract |
|  | A pilot study of dimeticone oils versus sodium carbonate treatment for tungiasis: A randomized cohort trial in Homa Bay County, Kenya | Gete B. & Leykun B. | April 3, 2024 | Title & abstract |
|  | A plant-based repellent protects against Tunga penetrans infestation and sand flea disease | Gete B. & Leykun B. | April 3, 2024 | Title & abstract |
|  | A practical approach to common skin problems in returning travellers | Gete B. & Leykun B. | April 3, 2024 | Title & abstract |
|  | A preliminary investigation of the flea control effect of beta-cypermethrin in some rural residential areas of Xinjiang Uygur autonomous region, China. | Gete B. & Leykun B. | April 3, 2024 | Title & abstract |
|  | A qualitative case study of community experiences with Tungiasis in high prevalence villages of Bungoma County, Kenya: | Gete B. & Leykun B. | April 3, 2024 | Title & abstract |
|  | A rapid appraisal of the efficacy of sodium carbonate in the management of Tunga penetrans infestation at Uuna Primary School in Karemo Division, Siaya District, Kenya | Gete B. & Leykun B. | April 3, 2024 | Title & abstract |
|  | A rapid assessment method for the estimation of the occurrence of epidermal parasitic skin diseases in Brazil: tungiasis and scabies as case studies | Gete B. & Leykun B. | April 3, 2024 | Title & abstract |
|  | A rapid assessment method to estimate the prevalence of ectoparasitoses in Brazil: tungiasis and scabies as case studies | Gete B. & Leykun B. | April 3, 2024 | Title & abstract |
|  | A retrospective study of ectoparasitosis in patients referred to Imam Reza Hospital of Mashhad, Iran | Gete B. & Leykun B. | April 3, 2024 | Title & abstract |
|  | A review of ectoparasites and their effect on cattle production | Gete B. & Leykun B. | April 3, 2024 | Title & abstract |
|  | A review of public health important fleas (Insecta, Siphonaptera) and flea-borne diseases in India. | Gete B. & Leykun B. | April 3, 2024 | Title & abstract |
|  | A review of public health important fleas (Insecta, Siphonaptera) and flea-borne diseases in India | Gete B.& Leykun B. | April 3, 2024 | Title & abstract |
|  | A review of the fleas (Insecta: Siphonaptera) from Argentina | Gete B. & Leykun B. | April 3, 2024 | Title & abstract |
|  | A review of the traditional use of southern African medicinal plants for the treatment of selected parasite infections affecting humans | Gete B. & Leykun B. | April 3, 2024 | Title & abstract |
|  | A review on skin infections among children and its prevalence in school environment | Gete B. & Leykun B. | April 3, 2024 | Title & abstract |
|  | A school survey of dermatological disorders and associated socio-economic factors in Lucknow; a region of north India | Gete B. & Leykun B. | April 3, 2024 | Title & abstract |
|  | A second species of Tunga (Insecta, Siphonaptera) infecting man: Tunga trimamillata | Gete B. & Leykun B. | April 3, 2024 | Title & abstract |
|  | A Self-medicating Attempt to Remove the Sand Flea from a Toe by a Young Chimpanzee | Gete B. & Leykun B. | April 3, 2024 | Title & abstract |
|  | A simple method for rapid community assessment of tungiasis. | Gete B. & Leykun B. | April 3, 2024 | Title & abstract |
|  | A sticktight flea removed from the cheek of a two-year-old boy from Los Angeles | Gete B. & Leykun B. | April 3, 2024 | Title & abstract |
|  | A study of dermatological disorders in relation to personal hygiene and nutritional indicators among govt. high school children of age group 11-16 yrs | Gete B. & Leykun B. | April 3, 2024 | Title & abstract |
|  | A study of the spectrum of skin disease occurring in a black population in south‐east London | Gete B. & Leykun B. | April 3, 2024 | Title & abstract |
|  | A subungual nodule of recent onset. Tungiasis | Gete B. & Leykun B. | April 3, 2024 | Title & abstract |
|  | A survey on Ectoparasites and haemoparasites of free-range indigenous chickens of Northern Tanzania | Gete B. & Leykun B. | April 3, 2024 | Title & abstract |
|  | A ten-year retrospective study of the prevalence of parasitic infections of dogs at the University of Maiduguri Veterinary Teaching Hospital, Nigeria | Gete B. & Leykun B. | April 3, 2024 | Title & abstract |
|  | A tourist with tungiasis | Gete B. & Leykun B. | April 3, 2024 | Title & abstract |
|  | A traveller's wart: tungiasis | Gete B. & Leykun B. | April 3, 2024 | Title & abstract |
|  | A walk in the woods with Tunga penetrans | Gete B. & Leykun B. | April 3, 2024 | Title & abstract |
|  | A way of measuring poverty that could further a change for the better | Gete B. & Leykun B. | April 3, 2024 | Title & abstract |
|  | Acne in schoolchildren: no longer a concern for dermatologists. | Gete B. & Leykun B. | April 3, 2024 | Title & abstract |
|  | Acquisition of yersinia murine toxin enabled Yersinia pestis to expand the range of mammalian hosts that sustain flea-borne plague | Gete B. & Leykun B. | April 3, 2024 | Title & abstract |
|  | Acute morbidity associated with scabies and other ectoparasitoses rapidly improves after treatment with ivermectin | Gete B. & Leykun B. | April 3, 2024 | Title & abstract |
|  | Additional description of a new species of Tunga (Siphonaptera) from Ecuador | Gete B. & Leykun B. | April 3, 2024 | Title & abstract |
|  | Adolescents with skin disease have specific quality of life issues | Gete B. & Leykun B. | April 3, 2024 | Title & abstract |
|  | Advances in tropical diseases | Gete B. & Leykun B. | April 3, 2024 | Title & abstract |
|  | Age and sex prevalence of infectious dermatoses among primary school children in a rural South-Eastern Nigerian community | Gete B. & Leykun B. | April 3, 2024 | Title & abstract |
|  | Age related, structured educational programmes for the management of atopic dermatitis in children and adolescents: multicentre, randomised controlled trial | Gete B. & Leykun B. | April 3, 2024 | Title & abstract |
|  | Air transportation of tungiasis | Gete B. & Leykun B. | April 3, 2024 | Title & abstract |
|  | Aleixo de Abreu [1568-1630], author of the earliest book on tropical medicine describing amoebiasis, malaria, typhoid fever, scurvy, yellow fever, dracontiasis, trichuriasis and tungiasis in 1623 | Gete B. & Leykun B. | April 3, 2024 | Title & abstract |
|  | An Ectopic Case of Tunga spp. Infection in Peru. | Gete B. & Leykun B. | April 3, 2024 | Title & abstract |
|  | An epidemiological study of prevalence of skin diseases among secondary school going children in District Meerut | Gete B. & Leykun B. | April 3, 2024 | Title & abstract |
|  | An exploration of factors associated with jigger infestation (Tungiasis) among residents of Muranga North District, Kenya. | Gete B. & Leykun B. | April 3, 2024 | Title & abstract |
|  | An instance of tungiasis in New Zealand | Gete B. & Leykun B. | April 3, 2024 | Title & abstract |
|  | An integrated active case detection and management of skin NTDs in yaws endemic health districts in Cameroon, Côte d'Ivoire and Ghana | Gete B. & Leykun B. | April 3, 2024 | Title & abstract |
|  | An outbreak of Tunga penetrans in a pig herd | Gete B. & Leykun B. | April 3, 2024 | Title & abstract |
|  | An overview of fleas (Siphonaptera) in wild and domestic mammals from Algeria with new data from the central north and south of the country. | Gete B. & Leykun B. | April 3, 2024 | Title & abstract |
|  | An unusual case of ectopic tungiasis with pseudoepitheliomatous hyperplasia. | Gete B. & Leykun B. | April 3, 2024 | Title & abstract |
|  | An unusual cause of plantar pustulosis | Gete B. & Leykun B. | April 3, 2024 | Title & abstract |
|  | Anatomy of Tunga trimamillata Pampiglione et al., 2002 (Insecta, Siphonaptera, Tungidae) and developmental phases of the gravid female | Gete B. & Leykun B. | April 3, 2024 | Title & abstract |
|  | Anemia, leukocytosis and eosinophilia in a resource-poor population with helmintho-ectoparasitic coinfection | Gete B. & Leykun B. | April 3, 2024 | Title & abstract |
|  | Animal and human tungiasis-related knowledge and treatment practices among animal keeping households in Bugiri District, South-Eastern Uganda. | Gete B. & Leykun B. | April 3, 2024 | Title & abstract |
|  | Animal Reservoirs of Zoonotic Tungiasis in Endemic Rural Villages of Uganda. | Gete B. & Leykun B. | April 3, 2024 | Title & abstract |
|  | Animal Tungiasis in the endemic areas of Badagry Local Government Area of Lagos State, Southwestern Nigeria | Gete B. & Leykun B. | April 3, 2024 | Title & abstract |
|  | Annual cycles of four flea species in the central Negev desert. | Gete B. & Leykun B. | April 3, 2024 | Title & abstract |
|  | Anthropogenic disturbance and the risk of flea-borne disease transmission. | Gete B. & Leykun B. | April 3, 2024 | Title & abstract |
|  | Anti-Jiggers pilot intervention program and rural health systems strengthening in Western Province, Kenya | Gete B. & Leykun B. | April 3, 2024 | Title & abstract |
|  | Antiparasitic activity of tea tree oil (TTO) and its components against medically important ectoparasites: A systematic review | Gete B. & Leykun B. | April 3, 2024 | Title & abstract |
|  | Approach to skin problems in travellers: clinical and epidemiological clues | Gete B. & Leykun B. | April 3, 2024 | Title & abstract |
|  | Arthropod bites, stings, and infestations: their prevention and treatment | Gete B. & Leykun B. | April 3, 2024 | Title & abstract |
|  | Arthropod-Associated Skin Diseases among Occupants of Five Designated Junior Staffs\'Quarters in Owerri and Enugu, Nigeria | Gete B. & Leykun B. | April 3, 2024 | Title & abstract |
|  | Arthropod-borne diseases associated with political and social disorder | Gete B. & Leykun B. | April 3, 2024 | Title & abstract |
|  | Arthropod-borne diseases in Italy: from a neglected matter to an emerging health problem. | Gete B. & Leykun B. | April 3, 2024 | Title & abstract |
|  | Arthropods in nests of the sand martin (Riparia riparia Linnaeus, 1758) in South Slovakia | Gete B. & Leykun B. | April 3, 2024 | Title & abstract |
|  | Assessing skin disease and associated health-related quality of life in a rural Lao community | Gete B. & Leykun B. | April 3, 2024 | Title & abstract |
|  | Assessing the impact of tungiasis on children's quality of life in Kenya | Gete B. & Leykun B. | April 3, 2024 | Title & abstract |
|  | Assessing the potentials of two local topical ointments as affordable treatment against tungiasis infestation: A self-experimentation in Igbokoda, Nigeria | Gete B. & Leykun B. | April 3, 2024 | Outcome |
|  | Assessment of factors influencing jigger infestation in Households with primary school going pupils in Karungu, Migori county, Kenya | Gete B. & Leykun B. | April 3, 2024 | Outcome |
|  | Assessment of pupils' knowledge and practices towards prevention and control of tungiasis infestation in Ugenya sub county, Kenya | Gete B. & Leykun B. | April 3, 2024 | Title & abstract |
|  | Assessment of socioeconomic status and the prevalence of Tungiasis in Jimma and Wolaita Sodo, Ethiopia | Gete B. & Leykun B., Belay D. | April 3, 2024 | Low quality |
|  | Assessment of the prevalence of podoconiosis and tungiasis in kibaale and kyenjojo districts of mid-western uganda | Gete B. & Leykun B. | April 3, 2024 | Outcome |
|  | Assessment of the role played by domestic animals in jigger infection in Kandara sub-county, Kenya (case control study) | Gete B. & Leykun B. | April 3, 2024 | Title & abstract |
|  | Assessment of the Role Played by Housing Conditions in Exacerbating Tungiasis in Kandara Sub-County, Kenya | Gete B. & Leykun B. | April 3, 2024 | Outcome |
|  | Assessment of the school environment for risk factors for tungiasis in nine counties of Kenya: a cross-sectional survey | Gete B. & Leykun B. | April 3, 2024 | Outcome |
|  | Assessment of tungiasis management knowledge in Kandara sub county, Kenya | Gete B. & Leykun B. | April 3, 2024 | Title & abstract |
|  | Association between footwear use and neglected tropical diseases: a systematic review and meta-analysis. | Gete B. & Leykun B. | April 3, 2024 | Title & abstract |
|  | Association between preschool eczema and medication for attention‐deficit/hyperactivity disorder in school age | Gete B. & Leykun B. | April 3, 2024 | Title & abstract |
|  | Association between skin disorders and depression in children and adolescents: A retrospective case-control study | Gete B. & Leykun B. | April 3, 2024 | Title & abstract |
|  | Association between Tungiasis, the Type of Housing and Environment: Evidence from Kitany Location, Keiyo Marakwet County, Kenya | Gete B. & Leykun B. | April 3, 2024 | Outcome |
|  | Association of antibiotics use in preschool age with atopic and allergic skin diseases in young adulthood: a population-based retrospective cohort study | Gete B. & Leykun B. | April 3, 2024 | Title & abstract |
|  | Associations between innate immune function and ectoparasites in wild rodent hosts | Gete B. & Leykun B. | April 3, 2024 | Title & abstract |
|  | Atopic dermatitis caused by ascariasis and cured with nitazoxanide | Gete B. & Leykun B. | April 3, 2024 | Title & abstract |
|  | Atopic disorders and parasitic infections | Gete B. & Leykun B. | April 3, 2024 | Title & abstract |
|  | Awareness of parasitic zoonotic diseases among pet owners in Cairo, Egypt | Gete B. & Leykun B. | April 3, 2024 | Title & abstract |
|  | Bacterial superinfection in human tungiasis. | Gete B. & Leykun B. | April 3, 2024 | Title & abstract |
|  | Baseline study on the Integrated Project on Family Planning, Parasite Control and Nutrition in Zambia | Gete B. & Leykun B. | April 3, 2024 | Title & abstract |
|  | Basic epidemiology of infectious diseases | Gete B. & Leykun B. | April 3, 2024 | Title & abstract |
|  | Bedside diagnostics in dermatology: Parasitic and noninfectious diseases | Gete B. & Leykun B. | April 3, 2024 | Title & abstract |
|  | Behavioural aspects of the ecology of the sand martin flea Ceratophyllus styx jordani Smit (Siphonaptera) | Gete B. & Leykun B. | April 3, 2024 | Title & abstract |
|  | Bidirectional relationships between psychological health and dermatological conditions in children | Gete B. & Leykun B. | April 3, 2024 | Title & abstract |
|  | Bioactive compounds coated 2D scaffold from seeds of Carica papaya for bacterial and parasitic skin infections | Gete B. & Leykun B. | April 3, 2024 | Title & abstract |
|  | Biocontrol of the cat flea, Ctenocephalides felis, by entomopathogenic nematodes and fungi. | Gete B. & Leykun B. | April 3, 2024 | Title & abstract |
|  | Bites and Infestations | Gete B. & Leykun B. | April 3, 2024 | Title & abstract |
|  | Blood‐feeding ectoparasites as developmental stressors: Does corticosterone mediate effects of mite infestation on nestling growth, immunity and energy availability? | Gete B. & Leykun B. | April 3, 2024 | Title & abstract |
|  | Blood-feeding of Tunga penetrans males | Gete B. & Leykun B. | April 3, 2024 | Title & abstract |
|  | Breakfast, lunch, and dinner sign: a hallmark of flea and bedbug bites | Gete B. & Leykun B. | April 3, 2024 | Title & abstract |
|  | Bullous tungiasis | Gete B. & Leykun B. | April 3, 2024 | Title & abstract |
|  | Bullying in persons with skin diseases | Gete B. & Leykun B. | April 3, 2024 | Title & abstract |
|  | Burden and determinants of scabies in Ethiopian school age children: A systematic review and meta-analysis with public health implications | Gete B. & Leykun B. | April 3, 2024 | Title & abstract |
|  | Burden of atopic dermatitis in adults and adolescents: a systematic literature review | Gete B. & Leykun B. | April 3, 2024 | Title & abstract |
|  | Burden of skin disease and associated socioeconomic status in Asia: a cross-sectional analysis from the Global Burden of Disease Study 1990-2017 | Gete B. & Leykun B. | April 3, 2024 | Title & abstract |
|  | Burden of skin disease and associated socioeconomic status in Europe: an ecologic study from the Global Burden of Disease Study 2017 | Gete B. & Leykun B. | April 3, 2024 | Title & abstract |
|  | Burden of Skin Disease—China, 1990− 2019 | Gete B. & Leykun B. | April 3, 2024 | Title & abstract |
|  | Burden of skin diseases | Gete B. & Leykun B. | April 3, 2024 | Title & abstract |
|  | Can fleas from dogs infected with canine visceral leishmaniasis transfer the infection to other mammals? | Gete B. & Leykun B. | April 3, 2024 | Title & abstract |
|  | Canine tungiasis: High prevalence in a tourist region in Bahia state, Brazil | Gete B. & Leykun B. | April 3, 2024 | Title & abstract |
|  | Carotenoid‐induced maternal effects interact with ectoparasite burden and brood size to shape the trade‐off between growth and immunity in nestling great tits | Gete B. & Leykun B. | April 3, 2024 | Title & abstract |
|  | Case of Phthiriasis palpebrarum with blepheroconjunctivitis | Gete B. & Leykun B. | April 3, 2024 | Title & abstract |
|  | Case report: an ectopic case of Tunga spp. Infection in Peru | Gete B. & Leykun B. | April 3, 2024 | Title & abstract |
|  | Case Report: Evidence of Tungiasis in the Amazon Rain Forest of Ecuador | Gete B. & Leykun B. | April 3, 2024 | Title & abstract |
|  | Case report: histopathological features of tungiasis in Peru | Gete B. & Leykun B. | April 3, 2024 | Title & abstract |
|  | Case report: infestation of tunga penetrans in villages near zomba central hospital | Gete B. & Leykun B. | April 3, 2024 | Title & abstract |
|  | Case Report: Severe Infestation with Tungiasis in a Coastal Community in Badagry Lagos, Nigeria. | Gete B. & Leykun B. | April 3, 2024 | Title & abstract |
|  | Cat flea (Ctenocephalides felisfelis) and Oriental cat flea (Ctenocephalides orientis) infestation as an emerging nuisance to human population | Gete B. & Leykun B. | April 3, 2024 | Title & abstract |
|  | Cat Flea Infestation, Evolution and Endosymbionts, and Community Awareness Towards Fleas and Flea-Borne Diseases | Gete B. & Leykun B. | April 3, 2024 | Title & abstract |
|  | Cat-scratch disease and Bartonella henselae infections in children | Gete B. & Leykun B. | April 3, 2024 | Title & abstract |
|  | CDLQI-based assessment of skin disorders among children: a study from Northern India | Gete B. & Leykun B. | April 3, 2024 | Title & abstract |
|  | Cellulitis Surrounding Protruding Calcified Mass on Lower Leg | Gete B. & Leykun B. | May 19,2024 | Title & abstract |
|  | Cercarial dermatitis in Europe: a new public health problem? | Gete B. & Leykun B. | May 19,2024 | Title & abstract |
|  | Changing paradigms in parasitic infections: common dermatological helminthic infections and cutaneous myiasis | Gete B. & Leykun B. | May 19,2024 | Title & abstract |
|  | Characteristics and prevalence of non-syndrome multiple supernumerary teeth: a retrospective study | Gete B. & Leykun B. | May 19,2024 | Title & abstract |
|  | Characterization of ectoparasites on dogs in the nucleus of urban expansion of Juiz de Fora, Minas Gerais, Brazil. | Gete B. & Leykun B. | May 19,2024 | Title & abstract |
|  | Characterization of Tunga penetrans antigens in selected epidemic areas in Murang'a county in Kenya. | Gete B. & Leykun B. | May 19,2024 | Title & abstract |
|  | Characterization of tungiasis infection and morbidity in Kenya reveals increase in disease burden during COVID-19 school closures | Gete B. & Leykun B. | May 19,2024 | Title & abstract |
|  | Chigoe infestation | Gete B. & Leykun B. | May 19,2024 | Title & abstract |
|  | Children with tungiasis in Kenya have poor school performance and quality of life | Gete B. & Leykun B. | May 19,2024 | Title & abstract |
|  | Children, pets, and disease | Gete B. & Leykun B. | May 19,2024 | Title & abstract |
|  | Children's perception scale of head lice infestation (CPS-HLI): Design and psychometrics | Gete B. & Leykun B. | May 19,2024 | Title & abstract |
|  | Chronic ulcers and myasis as ports of entry for Clostridium tetani | Gete B. & Leykun B. | May 19,2024 | Title & abstract |
|  | Chronic urticaria revealing a colonic adenocarcinoma | Gete B. & Leykun B. | May 19,2024 | Title & abstract |
|  | Claw histopathology and parasitic load in natural cases of canine leishmaniosis associated with Leishmania infantum | Gete B. & Leykun B. | May 19,2024 | Title & abstract |
|  | Climate crises and developing vector-borne diseases: a narrative review | Gete B. & Leykun B. | May 19,2024 | Title & abstract |
|  | Clinical and epidemiological survey of tungiasis in Madagascar | Gete B. & Leykun B. | May 19,2024 | Title & abstract |
|  | Clinical and histopathologic study of 39 patients with imported tungiasis | Gete B. & Leykun B. | May 19,2024 | Title & abstract |
|  | Clinical and macroscopic morphological features of canine tungiasis | Gete B. & Leykun B. | May 19,2024 | Title & abstract |
|  | Clinical and pathological aspects of first report of Tunga penetrans infestation on southern brown howler monkey (Alouatta guariba clamitans) in Rio Grande do Sul, Brazil | Gete B. & Leykun B. | May 19,2024 | Title & abstract |
|  | Clinical implications and treatment options of tungiasis in domestic animals | Gete B. & Leykun B. | May 19,2024 | Title & abstract |
|  | Clinical interventions for tungiasis (sand flea disease): a systematic review | Gete B. & Leykun B. | May 19,2024 | Title & abstract |
|  | Clinical Pattern of Dermatological Conditions among Paediatric Patients Attending Outpatient Department in a Tertiary Care Centre of Rural Haryana, India. | Gete B. & Leykun B. | May 19,2024 | Title & abstract |
|  | Clinical Profile, Risk Factors and Outcomes of Children With Cutaneous Larva Migrans Infection: A Hospital-Based Study | Gete B. & Leykun B. | May 19,2024 | Title & abstract |
|  | Clinical, laboratory, and epidemiologic features of murine typhus in 97 Texas children | Gete B. & Leykun B. | May 19,2024 | Title & abstract |
|  | Clinico-epidemiological studies in canine dermatitis | Gete B. & Leykun B. | May 19,2024 | Title & abstract |
|  | Clinico-haemato biochemical findings in metazoan dermatitis in canines. | Gete B. & Leykun B. | May 19,2024 | Title & abstract |
|  | Clinico-mycological profile of dermatophytosis in a reference centre for leprosy and dermatological diseases in Addis Ababa | Gete B. & Leykun B. | May 19,2024 | Title & abstract |
|  | Common cutaneous parasites | Gete B. & Leykun B. | May 19,2024 | Title & abstract |
|  | Common Dermatologic Conditions in Returning Travelers | Gete B. & Leykun B. | May 19,2024 | Title & abstract |
|  | Common misdiagnoses and prevalence of dermatological disorders at a pediatric tertiary care center | Gete B. & Leykun B. | May 19,2024 | Title & abstract |
|  | Common skin problems | Gete B. & Leykun B. | May 19,2024 | Title & abstract |
|  | Community dermatology in Debre Markos: an attempt to define children's dermatological needs in a rural area of Ethiopia | Gete B. & Leykun B. | May 19,2024 | Title & abstract |
|  | Comparative dermatology: parasitic disorders | Gete B. & Leykun B. | May 19,2024 | Title & abstract |
|  | Complex interactions within the ectoparasite community of the eastern rock sengi (Elephantulus myurus) | Gete B. & Leykun B. | May 19,2024 | Title & abstract |
|  | Concern Raised Over Jigger-Infestation In The Country. Kenya News. | Gete B. & Leykun B. | May 19,2024 | Title & abstract |
|  | Consequences of nest reuse for parasite burden and female health and condition in blue tits, Cyanistes caeruleus | Gete B. & Leykun B. | May 19,2024 | Title & abstract |
|  | Contemporary approaches to jigger flea control in the pastoralist environment of the Maasai Community in Hai District, Kilimanjaro Region, Tanzania | Gete B. & Leykun B. | May 19,2024 | Title & abstract |
|  | Contraception is the best kept secret for prevention of mother-to-child HIV transmission | Gete B. & Leykun B. | May 19,2024 | Title & abstract |
|  | Contributions of socio-economic and cultural factors in Tungiasis at Kwakombo village in Korogwe district, Tanzania | Gete B. & Leykun B. | May 19,2024 | Title & abstract |
|  | Control of tungiasis in absence of a roadmap: grassroots and global approaches | Gete B. & Leykun B. | May 19,2024 | Title & abstract |
|  | Control of tungiasis through intermittent application of a plant-based repellent: an intervention study in a resource-poor community in Brazil | Gete B. & Leykun B. | May 19,2024 | Title & abstract |
|  | Control or elimination: terms for public health interventions against tungiasis and schistosomiasis haematobium | Gete B. & Leykun B. | May 19,2024 | Title & abstract |
|  | Controlling phlebotomine sand flies to prevent canine Leishmania infantum infection: A case of knowing your enemy | Gete B. & Leykun B. | May 19,2024 | Title & abstract |
|  | Controlling tungiasis in an impoverished community: an intervention study. | Gete B. & Leykun B. | May 19,2024 | Title & abstract |
|  | Correction: Characterization of Tunga penetrans antigens in selected epidemic areas in Murang'a County in Kenya. | Gete B. & Leykun B. | May 19,2024 | Title & abstract |
|  | Cost-Effective PCR-Based Identification of Tunga penetrans (Siphonaptera) Larvae Extracted from Soil Samples Containing PCR Inhibitor-Rich Material | Gete B. & Leykun B. | May 19,2024 | Title & abstract |
|  | Counting the burden: atopic dermatitis and health-related quality of life | Gete B. & Leykun B. | May 19,2024 | Title & abstract |
|  | Current status of the knowledge on the epidemiology of tungiasis in the Americas | Gete B. & Leykun B. | May 19,2024 | Title & abstract |
|  | Current Status of Tungiasis in Endemic Areas. Prevalence, Risk Factors, Prevention | Gete B. & Leykun B. | May 19,2024 | Title & abstract |
|  | Current tropical dermatoses. Tungiasis, tropical ulcer, leishmaniasis. Observations from general practice. | Gete B. & Leykun B. | May 19,2024 | Title & abstract |
|  | Current tropical dermatoses. Tungiasis, ulcus tropicum, leishmaniasis. Case observations | Gete B. & Leykun B. | May 19,2024 | Title & abstract |
|  | Cutaneous ectoparasites | Gete B. & Leykun B. | May 19,2024 | Title & abstract |
|  | Cutaneous furuncle-like lesions with an unexpected diagnosis: a case report | Gete B. & Leykun B. | May 19,2024 | Title & abstract |
|  | Cutaneous infestation by Tunga penetrans | Gete B. & Leykun B. | May 19,2024 | Title & abstract |
|  | Cutaneous larva migrans and tungiasis in international travelers exiting Brazil: an airport survey | Gete B. & Leykun B. | May 19,2024 | Title & abstract |
|  | Cutaneous larva migrans and tungiasis: infestations of the affluent society | Gete B. & Leykun B. | May 19,2024 | Title & abstract |
|  | Cutaneous larva migrans and tungiasis: the challenge to control zoonotic ectoparasitoses associated with poverty | Gete B. & Leykun B. | May 19,2024 | Title & abstract |
|  | Cutaneous Larva Migrans Infestation Over Buttocks and Perineal Region: A Case Series of Five Toddlers From Sri Lanka and Literature Review | Gete B. & Leykun B. | May 19,2024 | Title & abstract |
|  | Cutaneous manifestations of selected parasitic infections in western Pacific and southeast Asian regions | Gete B. & Leykun B. | May 19,2024 | Title & abstract |
|  | Cytopathology of parasitic dermatitis in dogs | Gete B. & Leykun B. | May 19,2024 | Title & abstract |
|  | Data on Demodex Ectoparasite Infestation in Patients Attending an Outpatient Clinic in Greece | Gete B. & Leykun B. | May 19,2024 | Title & abstract |
|  | Depression and skin disease | Gete B. & Leykun B. | May 19,2024 | Title & abstract |
|  | Dermatitis caused by autochthonous Cercopithifilaria bainae from a dog in Florida, USA: clinical, histological and parasitological diagnosis and treatment | Gete B. & Leykun B. | May 19,2024 | Title & abstract |
|  | Dermatitis rhabditidosa in an 11‐year‐old girl: a new cutaneous parasitic disease of man | Gete B. & Leykun B. | May 19,2024 | Title & abstract |
|  | Dermatologic conditions in teenage adolescents in Nigeria | Gete B. & Leykun B. | May 19,2024 | Title & abstract |
|  | Dermatologic conditions of the ill returned traveler: an analysis from the GeoSentinel Surveillance Network | Gete B. & Leykun B. | May 19,2024 | Title & abstract |
|  | Dermatologic diseases presenting with pigmentation disorders in children: a single center experience | Gete B. & Leykun B. | May 19,2024 | Title & abstract |
|  | Dermatologic Infectious Diseases in International Travelers | Gete B. & Leykun B. | May 19,2024 | Title & abstract |
|  | Dermatologic Manifestations of Parasitic Diseases | Gete B. & Leykun B. | May 19,2024 | Title & abstract |
|  | Dermatological conditions in international pediatric travelers: epidemiology, prevention and managemen | Gete B. & Leykun B. | May 19,2024 | Title & abstract |
|  | Dermatological Disorders amongst Primary School Children in Riyom Community, North-Central Nigeria | Gete B. & Leykun B. | May 19,2024 | Title & abstract |
|  | Dermatological disorders and dermatology-specific quality of life among secondary students in public and private schools in Kwara state, Nigeria | Gete B. & Leykun B. | May 19,2024 | Title & abstract |
|  | Dermatological disorders in children: a prospective analysis of 4025 patients | Gete B. & Leykun B. | May 19,2024 | Title & abstract |
|  | Dermatoscopy in inflammatory and infectious skin disorders | Gete B. & Leykun B. | May 19,2024 | Title & abstract |
|  | Dermatoscopy of parasitic and infectious disorders | Gete B. & Leykun B. | May 19,2024 | Title & abstract |
|  | Dermatoses associated with travel to tropical countries: a prospective study of the diagnosis and management of 269 patients presenting to a tropical disease unit | Gete B. & Leykun B. | May 19,2024 | Title & abstract |
|  | Dermoscopic features of tungiasis. | Gete B. & Leykun B. | May 19,2024 | Title & abstract |
|  | Dermoscopy in the diagnosis of tungiasis | Gete B. & Leykun B. | May 19,2024 | Title & abstract |
|  | Dermoscopy in tungiasis. | Gete B. & Leykun B. | May 19,2024 | Title & abstract |
|  | Dermoscopy of skin infestations and infections (entomodermoscopy) - Part I: dermatozoonoses and bacterial infections | Gete B. & Leykun B. | May 19,2024 | Title & abstract |
|  | Dermoscopy of tungiasis | Gete B. & Leykun B. | May 19,2024 | Title & abstract |
|  | Dermoscopy: Ex vivo visualization of fleas head and bag of eggs confirms the diagnosis of Tungiasis | Gete B. & Leykun B. | May 19,2024 | Title & abstract |
|  | Description of Tunga bonneti n. sp. from Chile (Siphonaptera: Tungidae) with notes on specificity, chorology, dermecos and phenology | Gete B. & Leykun B. | May 19,2024 | Title & abstract |
|  | Descriptions of three species of sand fleas (amphipods) collected at Newport, Rhode Island | Gete B. & Leykun B. | May 19,2024 | Title & abstract |
|  | Detection of a Neglected Tropical Disease: Case report of Heavy Tunga penetrans Lesions on the Feet of a Thirteen-Year-Old Boy from a Community in Lagos | Gete B. & Leykun B. | May 19,2024 | Title & abstract |
|  | Determinants of dermatological disorders among school children in Sagamu, Nigeria | Gete B. & Leykun B. | May 19,2024 | Title & abstract |
|  | Determinants Of Jigger Infestation: Knowledge, Attitude And Practices Among Household Members In Kilifi County, Kenya. | Gete B. & Leykun B. | May 19,2024 | Title & abstract |
|  | Determining the prevalence of schistosomiasis haematobium in population-groups from northern Tanzania based on the discordance between egg counts and haematuria. | Gete B. & Leykun B. | May 19,2024 | Title & abstract |
|  | Developing low-cost house floors to control tungiasis in Kenya–a feasibility study | Gete B. & Leykun B. | May 19,2024 | Title & abstract |
|  | Diabetes mellitus and travel-related illnesses | Gete B. & Leykun B. | May 19,2024 | Title & abstract |
|  | Diagnosis and treatment of a parasitic dermatitis in a laboratory colony of African clawed frogs (Xenopus laevis) | Gete B. & Leykun B. | May 19,2024 | Title & abstract |
|  | Differential diagnosis between Tunga penetrans (L., 1758) and T. trimamillata Pampiglione et al., 2002 (Insecta, Siphonaptera), the two species of the genus Tunga parasitic in man | Gete B. & Leykun B. | May 19,2024 | Title & abstract |
|  | Dipylidium caninum infection in a 14-month-old child. | Gete B. & Leykun B. | May 19,2024 | Title & abstract |
|  | Dipylidium caninum infection in a child: a rare case report | Gete B. & Leykun B. | May 19,2024 | Title & abstract |
|  | Dipylidium caninum Infection in Dogs and Humans in Bishoftu Town, Ethiopia | Gete B. & Leykun B. | May 19,2024 | Title & abstract |
|  | Direct injury: phobias, psychoses, annoyance, allergies, toxins, venoms and myiasis | Gete B. & Leykun B. | May 19,2024 | Title & abstract |
|  | Dirt, worms and atopic dermatitis | Gete B. & Leykun B. | May 19,2024 | Title & abstract |
|  | Disease co-morbidity and the human Wnt signaling pathway: a network-wise study | Gete B. & Leykun B. | May 19,2024 | Title & abstract |
|  | Diseases caused by arthropods and other noxious animals | Gete B. & Leykun B. | May 19,2024 | Title & abstract |
|  | Diseases caused by arthropods-moth larva in clitoris, tungiasis, cutaneous and intestinal myiasis in Russian travelers: report of eight cases and review the literature | Gete B. & Leykun B. | May 19,2024 | Title & abstract |
|  | Disseminated tungiasis | Gete B. & Leykun B. | May 19,2024 | Title & abstract |
|  | Disseminated tungiasis in a 78-year-old woman from Tanzania: a case report. | Gete B. & Leykun B. | May 19,2024 | Title & abstract |
|  | Disseminated tungiasis in an 8-year-old girl | Gete B. & Leykun B. | May 19,2024 | Title & abstract |
|  | Distribution and prevalence of Tunga penetrans in coastal south Trinidad, West Indies | Gete B. & Leykun B. | May 19,2024 | Title & abstract |
|  | Distribution of tungiasis in latin America: Identification of areas for potential disease transmission using an ecological niche model. | Gete B. & Leykun B. | May 19,2024 | Title & abstract |
|  | Distribution patterns of Tunga penetrans within a community in Trinidad, West Indies | Gete B. & Leykun B. | May 19,2024 | Title & abstract |
|  | Do intestinal parasites enhance food allergy and atopic dermatitis?: A study in Warao Amerindian children | Gete B. & Leykun B. | May 19,2024 | Title & abstract |
|  | Does coconut oil aid in improving the outcome of dermatologic conditions, particularly acute tungiasis and atopic dermatitis, by improving lesions and lessening | Gete B. & Leykun B. | May 19,2024 | Title & abstract |
|  | Does parasitic infection protect against allergy? | Gete B. & Leykun B. | May 19,2024 | Title & abstract |
|  | Dog tapeworm (Dipylidium caninum) infestation in a 6-month-old infant | Gete B. & Leykun B. | May 19,2024 | Title & abstract |
|  | Drivers of the Ectoparasite community and co-infection patterns in rural and urban burrowing owls | Gete B. & Leykun B. | May 19,2024 | Title & abstract |
|  | Dynamic behavior of a stochastic tungiasis model for public health education | Gete B. & Leykun B. | May 19,2024 | Title & abstract |
|  | Dynamics and optimal control in transmission of tungiasis diseases | Gete B. & Leykun B. | May 19,2024 | Title & abstract |
|  | EADV Taskforce's recommendations on measurement of health‐related quality of life in paediatric dermatology | Gete B. & Leykun B. | May 19,2024 | Title & abstract |
|  | Early references to the occurrence of Tunga penetrans in tropical Africa. | Gete B. & Leykun B. | May 19,2024 | Title & abstract |
|  | Ecological correlates of ectoparasite load in a rodent: Complex roles of seasonality | Gete B. & Leykun B. | May 19,2024 | Title & abstract |
|  | Ectoparasite burden influences the denning behavior of a small desert carnivore | Gete B. & Leykun B. | May 19,2024 | Title & abstract |
|  | Ectoparasite burden, clinical disease, and immune responses throughout fur mite (Myocoptes musculinus) infestation in C57BL/6 and Rag1–/–mice | Gete B. & Leykun B. | May 19,2024 | Title & abstract |
|  | Ectoparasite burden, clinical disease, and immune responses throughout fur mite (Myocoptes musculinus) infestation in C57BL/6 and Rag1–/–mice | Gete B. & Leykun B. | May 19,2024 | Title & abstract |
|  | Ectoparasite Burdens of the Common Mole-Rat (Cryptomys hottentotus hottentotus) From the Cape Provinces of South Africa | Gete B. & Leykun B. | May 19,2024 | Title & abstract |
|  | Ectoparasite Burdens of the Damaraland Mole-Rat (Fukomys damarensis) from Southern Africa | Gete B. & Leykun B. | May 19,2024 | Title & abstract |
|  | Ectoparasite impacts on Gerbillus andersoni allenbyi under natural conditions | Gete B. & Leykun B. | May 19,2024 | Title & abstract |
|  | Ectoparasite Infestation Of Goats (Capra Hircus) In Northern Iraq: Prevalence And Species Identification | Gete B. & Leykun B. | May 19,2024 | Title & abstract |
|  | Ectoparasite intensities are correlated with endoparasite infection loads in willow ptarmigan | Gete B. & Leykun B. | May 19,2024 | Title & abstract |
|  | Ectoparasite load increase in reproductively active sand lizards | Gete B. & Leykun B. | May 19,2024 | Title & abstract |
|  | Ectoparasite load of small mammals in the Serengeti Ecosystem: effects of land use, season, host species, age, sex and breeding status | Gete B. & Leykun B. | May 19,2024 | Title & abstract |
|  | Ectoparasite loads in sympatric urban populations of the northern white-breasted and the European hedgehog | Gete B. & Leykun B. | May 19,2024 | Title & abstract |
|  | Ectoparasites and age-dependent survival in a desert rodent | Gete B. & Leykun B. | May 19,2024 | Title & abstract |
|  | Ectoparasites and Arthropod Vectors: Ectoparasite Infestations | Gete B. & Leykun B. | May 19,2024 | Title & abstract |
|  | Ectoparasites and haemoparasites of indigenous chicken (gallus domesticus) in Ibadan and environs. | Gete B. & Leykun B. | May 19,2024 | Title & abstract |
|  | Ectoparasites and sett use in European badgers | Gete B. & Leykun B. | May 19,2024 | Title & abstract |
|  | Ectoparasites and their damage in backyard turkeys in Oaxaca's coast, Mexico | Gete B. & Leykun B. | May 19,2024 | Title & abstract |
|  | Ectoparasites and their effect on camels (Camelus dromedarius) in Ethiopia | Gete B. & Leykun B. | May 19,2024 | Title & abstract |
|  | Ectoparasites as developmental stressors: Effects on somatic and physiological development | Gete B. & Leykun B. | May 19,2024 | Title & abstract |
|  | Ectoparasites burden of House mouse (Mus musculus linnaeus, 1758) from Hai'l region, Kingdom of Saudi Arabia | Gete B. & Leykun B. | May 19,2024 | Title & abstract |
|  | Ectoparasites do not affect ejaculate volume in the dragonfly Coenagrion puella | Gete B. & Leykun B. | May 19,2024 | Title & abstract |
|  | Ectoparasites Host Resistance and Tolerance | Gete B. & Leykun B. | May 19,2024 | Title & abstract |
|  | Ectoparasites in small exotic mammals | Gete B. & Leykun B. | May 19,2024 | Title & abstract |
|  | Ectoparasites of buffaloes (Bubalus bubalis) in Kurigram district of Bangladesh | Gete B. & Leykun B. | May 19,2024 | Title & abstract |
|  | Ectoparasites of dogs (Canis familiaris) from northeastern Nigeria: an epidemiological study | Gete B. & Leykun B. | May 19,2024 | Title & abstract |
|  | Ectoparasites of dogs and cats, their importance and distribution in Europe | Gete B. & Leykun B. | May 19,2024 | Title & abstract |
|  | Ectoparasites of dogs belonging to people in resource-poor communities in North West Province, South Africa | Gete B. & Leykun B. | May 19,2024 | Title & abstract |
|  | Ectoparasites of endemic and domestic animals in southwest Madagascar | Gete B. & Leykun B. | May 19,2024 | Title & abstract |
|  | Ectoparasites of European hedgehogs (Erinaceus europaeus) in Germany and their health impact | Gete B. & Leykun B. | May 19,2024 | Title & abstract |
|  | Ectoparasites of free-roaming domestic cats in the central United States | Gete B. & Leykun B. | May 19,2024 | Title & abstract |
|  | Ectoparasites of livestock in Europe and the Mediterranean region | Gete B. & Leykun B. | May 19,2024 | Title & abstract |
|  | Ectoparasites of livestock, dogs, and wild rodents in the Chittagong Hill Tracts in southeastern Bangladesh | Gete B. & Leykun B. | May 19,2024 | Title & abstract |
|  | Ectoparasites of murids in peninsular Malaysia and their associated diseases | Gete B. & Leykun B. | May 19,2024 | Title & abstract |
|  | Ectoparasites of rodents captured in Bandar Abbas, southern Iran | Gete B. & Leykun B. | May 19,2024 | Title & abstract |
|  | Ectoparasites of stray cats in Bangkok metropolitan areas, Thailand | Gete B. & Leykun B. | May 19,2024 | Title & abstract |
|  | Ectoparasites on small mammals in Iceland: Origin and population characteristics of a species‐poor insular community | Gete B. & Leykun B. | May 19,2024 | Title & abstract |
|  | Ectoparasites prevalence in small ruminants in and around Sekela, Amhara Regional State, Northwest Ethiopia | Gete B. & Leykun B. | May 19,2024 | Title & abstract |
|  | Ectoparasites. Part 2: Bed bugs, Demodex, sand fleas and cutaneous larva migrans. | Gete B. & Leykun B. | May 19,2024 | Title & abstract |
|  | Ectoparasites: future challenges in a changing world | Gete B. & Leykun B. | May 19,2024 | Title & abstract |
|  | Ectoparasites: Myiasis, Tungiasis, Scabies | Gete B. & Leykun B. | May 19,2024 | Title & abstract |
|  | Ectoparasites: pediculosis and tungiasis | Gete B. & Leykun B. | May 19,2024 | Title & abstract |
|  | Ectoparasites--the underestimated realm | Gete B. & Leykun B. | May 19,2024 | Title & abstract |
|  | Ectoparasitic and endoparasitic drug delivery approaches for therapy | Gete B. & Leykun B. | May 19,2024 | Title & abstract |
|  | Ectoparasitic community of the Mahali mole-rat, Cryptomys hottentotus mahali: potential host for vectors of medical importance in South Africa | Gete B. & Leykun B. | May 19,2024 | Title & abstract |
|  | Ectoparasitic fauna of poultry species in Maiduguri, Borno State, Nigeria | Gete B. & Leykun B. | May 19,2024 | Title & abstract |
|  | Ectoparasitic infestations | Gete B. & Leykun B. | May 19,2024 | Title & abstract |
|  | Ectoparasitic infestations in homeless children | Gete B. & Leykun B. | May 19,2024 | Title & abstract |
|  | Ectoparasitic infestations of the eye: Three cases with three different arthropods | Gete B. & Leykun B. | May 19,2024 | Title & abstract |
|  | Ectoparasitic infestations. Current Infectious Disease Reports | Gete B. & Leykun B. | May 19,2024 | Title & abstract |
|  | Ectoparasitism and infections in the exoskeletons of large fossil cingulates | Gete B. & Leykun B. | May 19,2024 | Title & abstract |
|  | Ectoparasitism as a possible cost of social life: a comparative analysis using Australian passerines (Passeriformes) | Gete B. & Leykun B. | May 19,2024 | Title & abstract |
|  | Ectopic knee tungiasis and historical aspects in Peru | Gete B. & Leykun B. | May 19,2024 | Title & abstract |
|  | Ectopic localization of tungiasis. | Gete B. & Leykun B. | May 19,2024 | Title & abstract |
|  | Editorial: Cutaneous larva migrans and tungiasis: the challenge to control zoonotic ectoparasitoses associated with poverty | Gete B. & Leykun B. | May 19,2024 | Title & abstract |
|  | Effect of jigger infestation on agricultural productivity: A case study of Murarandia Division | Gete B. & Leykun B. | May 19,2024 | Title & abstract |
|  | Effectiveness of 20% salicylated vaseline in the treatment of profuse tungiasis. Report of 8 cases in French Guiana | Gete B. & Leykun B. | May 19,2024 | Title & abstract |
|  | Effectiveness of an integrated intervention in the control of endo-and ectoparasites of pigs kept by smallholder farmers in Mbeya rural and Mbozi districts, Tanzania | Gete B. & Leykun B. | May 19,2024 | Title & abstract |
|  | Effects Of Ectoparasitism On Host Condition In The Drosophila–Macrocheles System | Gete B. & Leykun B. | May 19,2024 | Title & abstract |
|  | Effects of haematophagous ectoparasites on nestling house wrens, Troglodytes aedon: who pays the cost of parasitism? | Gete B. & Leykun B. | May 19,2024 | Title & abstract |
|  | Effects of household vectors on child health and its determinants in southwest, Ethiopia correspondence analysis | Gete B. & Leykun B. | May 19,2024 | Title & abstract |
|  | Effects of Social Media in Controlling Tungiasis: Mathematical Model | Gete B. & Leykun B. | May 19,2024 | Title & abstract |
|  | Efficacy and safety of dimeticones in the treatment of epidermal parasitic skin diseases with special emphasis on tungiasis: an evidence-based critical review. | Gete B. & Leykun B. | May 19,2024 | Title & abstract |
|  | Efficacy of a mixture of neem seed oil (Azadirachta indica) and coconut oil (Cocos nucifera) for topical treatment of tungiasis. A randomized controlled, proof-of-principle study | Gete B. & Leykun B. | May 19,2024 | Title & abstract |
|  | Efficacy of an oral formulation of afoxolaner and milbemycin oxime against Tunga penetrans in naturally infected dogs. | Gete B. & Leykun B. | May 19,2024 | Title & abstract |
|  | Efficacy of coconut oil in control of tungiases | Gete B. & Leykun B. | May 19,2024 | Title & abstract |
|  | Efficacy of Coconut Oil in the Control of Acute Tungiasis | Gete B. & Leykun B. | May 19,2024 | Title & abstract |
|  | Efficacy of *Ivermectin* in a patient population concomitantly infected with intestinal helminths and ectoparasites | Gete B. & Leykun B. | May 19,2024 | Title & abstract |
|  | Efficacy of monthly treatment with oral fluralaner (Bravecto() 1-Month) against Tunga penetrans in dogs in Brazil: a randomized, double-blind, controlled field study | Gete B. & Leykun B. | May 19,2024 | Title & abstract |
|  | Efficacy of oral fluralaner (Bravecto) against Tunga penetrans in dogs: A negative control, randomized field study in an endemic community in Brazil | Gete B. & Leykun B. | May 19,2024 | Title & abstract |
|  | Efficacy of various insecticides in the environmental control of Tunga penetrans (L., 1758). | Gete B. & Leykun B. | May 19,2024 | Title & abstract |
|  | Emerging arthropod-borne diseases of companion animals in Europe | Gete B. & Leykun B. | May 19,2024 | Title & abstract |
|  | Emerging imported parasitoses in Italy | Gete B. & Leykun B. | May 19,2024 | Title & abstract |
|  | Emerging infectious diseases of the skin: a review of clinical and histologic findings | Gete B. & Leykun B. | May 19,2024 | Title & abstract |
|  | Energetic costs of ectoparasite infection in Atlantic salmon | Gete B. & Leykun B. | May 19,2024 | Title & abstract |
|  | Enhancing public health strategies for Tungiasis: A mathematical approach with fractional derivative | Gete B. & Leykun B. | May 19,2024 | Title & abstract |
|  | Entirely extracted Tunga penetrans in a traveller to Guinea-Bissau | Gete B. & Leykun B. | May 19,2024 | Title & abstract |
|  | Entodermoscopy: a new tool for diagnosing skin infections and infestations | Gete B. & Leykun B. | May 19,2024 | Title & abstract |
|  | Environmental and Household-Based Spatial Risks for Tungiasis in an Endemic Area of Coastal Kenya. | Gete B. & Leykun B. | May 19,2024 | Outcome |
|  | Environmental factors predisposing rural community members to Tungiasis in murangâ a east sub county, murangâ a county | Gete B. & Leykun B. | May 19,2024 | Title & abstarct |
|  | Environmental Parasitic Disease Affecting the Health Status in Rwanda | Gete B. & Leykun B. | May 19,2024 | Title & abstract |
|  | Epidemics of tungiasis in Rio Grande do Sul. | Gete B. & Leykun B. | May 19,2024 | Title & abstract |
|  | Epidemiologic features and burden of atopic dermatitis in adolescent and adult patients: A cross-sectional multicenter study | Gete B. & Leykun B. | March 18, 2024 | Title & abstract |
|  | Epidemiological and prophylactic aspects of tungiasis in dogs from Jataí, State of Goiás, Brazil | Gete B. & Leykun B. | March 18, 2024 | Title & abstract |
|  | Epidemiological aspects of head lice in children attending day care centres, urban and rural schools in Uberlandia, central Brazil | Gete B. & Leykun B. | March 18, 2024 | Title & abstract |
|  | Epidemiological studies on parasitic dermatitis in canines. | Gete B. & Leykun B. | March 18, 2024 | Title & abstract |
|  | Epidemiology and clinical aspects of tungiasis (sand flea infestation) in Alagoas State, Brazil | Gete B. & Leykun B. | March 18, 2024 | Title & abstract |
|  | Epidemiology and management of common skin diseases in children in developing countries | Gete B. & Leykun B. | March 18, 2024 | Title & abstract |
|  | Epidemiology and pathology of ectoparasitic infestations in black Bengal goats in Gaibandha and mymensingh districts of Bangladesh | Gete B. & Leykun B. | March 18, 2024 | Title & abstract |
|  | Epidemiology of cat-scratch disease hospitalizations among children in the United States | Gete B. & Leykun B. | March 18, 2024 | Title & abstract |
|  | Epidemiology of Common Parasitic Infections of the Skin In Infants and Children | Gete B. & Leykun B. | March 18, 2024 | Title & abstract |
|  | Epidemiology of dermatophyte infections among school children in Menoufia Governorate, Egypt | Gete B. & Leykun B. | March 18, 2024 | Title & abstract |
|  | Epidemiology of dermatoses in children and adolescents in Punjab, India. | Gete B. & Leykun B. | March 18, 2024 | Title & abstract |
|  | Epidemiology of ectoparasitic infestations in cattle at Bhawal forest area, Gazipur. | Gete B. & Leykun B. | March 18, 2024 | Title & abstract |
|  | Epidemiology of porcine and human tungiasis in Matadi (Zaire) and its economic and hygienic importance | Gete B. & Leykun B. | March 18, 2024 | Title & abstract |
|  | Epidemiology of skin disease | Gete B. & Leykun B. | March 18, 2024 | Title & abstract |
|  | Epidemiology of skin diseases in ethnic populations | Gete B. & Leykun B. | March 18, 2024 | Title & abstract |
|  | Epidemiology of skin diseases in school children: a study from northern India | Gete B. & Leykun B. | March 18, 2024 | Title & abstract |
|  | Epidemiology of skin disorders in Ethiopian children and adolescents: an analysis of records from the Italian Dermatological Centre, Mekelle, Tigray, Ethiopia, 2005 to | Gete B. & Leykun B. | March 18, 2024 | Title & abstract |
|  | Epidemiology of Tunga penetrans Infestation and Antigens Characterization in Selected Locations in Kiharu Constituency, Murang’a County, Kenya | Gete B. & Leykun B. | March 18, 2024 | Included |
|  | Epidemiology of tungiasis in sub-saharan Africa: a systematic review and meta-analysis. | Gete B. & Leykun B. | March 18, 2024 | Title & abstract |
|  | Epidermal parasitic skin diseases: a neglected category of poverty-associated plagues | Gete B. & Leykun B. | March 18, 2024 | Title & abstract |
|  | Erratum to "Tungiasis: more than an exotic nuisance" [Travel Medicine and Infectious Disease, 1 (2003) 159-166] | Gete B. & Leykun B. | March 18, 2024 | Title & abstract |
|  | Essential oils as a potential treatment option for pediculosis | Gete B. & Leykun B. | March 18, 2024 | Title & abstract |
|  | Establishment of Tunga trimamillata (Siphonaptera: Tungidae) in Brazil | Gete B. & Leykun B. | March 18, 2024 | Title & abstract |
|  | Estimation of point prevalence of common skin diseases affecting schoolchildren in the age group of 6–15 years in Jalandhar district of Punjab | Gete B. & Leykun B. | March 18, 2024 | Title & abstract |
|  | Ethnobotanical study of plants used in control of ectoparasites in Murang'a county, Kenya | Gete B. & Leykun B. | March 18, 2024 | Title & abstract |
|  | Evaluating the potentials of randomised integrated control trial on tungiasis in a South-western Nigerian community | Gete B. & Leykun B. | March 18, 2024 | Title & abstract |
|  | Evaluation of cases with Fasciola hepatica infection: experience in 6 children | Gete B. & Leykun B. | March 18, 2024 | Title & abstract |
|  | Evaluation of the lure and kill potential of sand paper leaves (Ficus exasperate) Against Fleas of Indigenous Fowls | Gete B. & Leykun B. | March 18, 2024 | Title & abstract |
|  | Evidence for double resistance to permethrin and malathion in head lice | Gete B. & Leykun B. | March 18, 2024 | Title & abstract |
|  | Evidence for interspecific interactions in the ectoparasite infracommunity of a wild mammal | Gete B. & Leykun B. | March 18, 2024 | Title & abstract |
|  | Evidence of tungiasis in pre-Hispanic America | Gete B. & Leykun B. | March 18, 2024 | Title & abstract |
|  | Evidence of Tungiasis in the Amazon Rain Forest of Ecuador. | Gete B. & Leykun B. | March 18, 2024 | Title & abstract |
|  | Exotic human myiasis. | Gete B. & Leykun B. | March 18, 2024 | Title & abstract |
|  | Experiences of appearance‐related teasing and bullying in skin diseases and their psychological sequelae: results of a qualitative study | Gete B. & Leykun B. | March 18, 2024 | Title & abstract |
|  | Experimental transmission of Bartonella henselae by the cat flea | Gete B. & Leykun B. | March 18, 2024 | Title & abstract |
|  | Exploring challenges and recommendations for verbal autopsy implementation in low-/middle-income countries: a cross-sectional study of Iringa Region-Tanzania | Gete B. & Leykun B. | March 18, 2024 | Title & abstract |
|  | Exploring healthcare professionals’ perspectives on neglected tropical diseases in Eastern Uganda: a qualitative study with a focus on schistosomiasis and soil-transmitted helminthes. | Gete B. & Leykun B. | March 18, 2024 | Title & abstract |
|  | Extended regorafenib treatment can be linked with mitochondrial damage leading to cardiotoxicity | Gete B. & Leykun B. | March 18, 2024 | Title & abstract |
|  | Exuberant Case of Tungiasis From Brazil | Gete B. & Leykun B. | March 18, 2024 | Title & abstract |
|  | Factors associated with diversity, quantity and zoonotic potential of ectoparasites on urban mice and voles | Gete B. & Leykun B. | March 18, 2024 | Title & abstract |
|  | Factors associated with tungiasis among primary school children: a cross-sectional study in a rural district in Rwanda | Gete B. & Leykun B. | March 18, 2024 | Included |
|  | Factors associated with tungiasis among school-age children in Kwale County, rural Kenya | Gete B. & Leykun B. | March 18, 2024 | Included |
|  | Factors Associated with Tungiasis Infestation among School age Children in Ugenya Sub-County, Siaya County, Kenya | Gete B. & Leykun B. | March 18, 2024 | Included |
|  | Factors contributing to prevalence of jigger infestation among community members of Mugumoini sub-location, Gatanga district Kenya Kenya Medical Training College. | Gete B. & Leykun B. | March 18, 2024 | Title & abstract |
|  | Factors influencing participation of stakeholders in eradication of jiggers: A case of Kandara sub county, Muranga county, Kenya | Gete B. & Leykun B. | March 18, 2024 | Included |
|  | Factors influencing practices towards water, sanitation and hygiene with occurrence of Tungiasis among pupils in schools with a feeding Programme in Ganze Sub County, Kenya | Gete B. & Leykun B. | March 18, 2024 | Outcome |
|  | Factors influencing prevention of Tungiasis infestation in Tshiatsala division of Butere Sub County–Kenya | Gete B. & Leykun B. | March 18, 2024 | Title & abstract |
|  | Feet deformities and their close association with postural stability deficits in children aged 10–15 years | Gete B. & Leykun B. | March 18, 2024 | Title & abstract |
|  | Field assessment of insecticide dusting and bait station treatment impact against rodent flea and house flea species in the Madagascar plague context | Gete B. & Leykun B. | March 18, 2024 | Title & abstract |
|  | Field studies on the behaviour of bird fleas: I. Behaviour of the adults of three species of bird fleas in the field | Gete B. & Leykun B. | March 18, 2024 | Title & abstract |
|  | Field trial of the efficacy of a combination of imidacloprid and permethrin against Tunga penetrans (sand flea, jigger flea) in dogs in Brazil | Gete B. & Leykun B. | March 18, 2024 | Title & abstract |
|  | First report of family infestation with pubic louse (Pthirus pubis; Insecta: Anoplura: Pthiridae) in Iran - a case report. | Gete B. & Leykun B. | March 18, 2024 | Title & abstract |
|  | First report of furuncular myiasis caused by the larva of botfly, Dermatobia hominis, in a Taiwanese traveler | Gete B. & Leykun B. | March 18, 2024 | Title & abstract |
|  | First surveys to investigate the presence of canine leishmaniasis and its phlebotomine vectors in Hungary | Gete B. & Leykun B. | March 18, 2024 | Title & abstract |
|  | Flat foot and associated factors among primary school children: A cross-sectional study | Gete B. & Leykun B. | March 18, 2024 | Title & abstract |
|  | Flea biology and control: the biology of the cat flea control and prevention with imidacloprid in small animals | Gete B. & Leykun B. | March 18, 2024 | Title & abstract |
|  | Flea bites and other diseases caused by fleas | Gete B. & Leykun B. | March 18, 2024 | Title & abstract |
|  | Flea Burden on Rodents and Its Associated Determinants in Plague‐Endemic Localities of Karatu District, Tanzania: A Cross‐Sectional Study | Gete B. & Leykun B. | March 18, 2024 | Title & abstract |
|  | Flea control: an overview of treatment concepts for North America | Gete B. & Leykun B. | March 18, 2024 | Title & abstract |
|  | Flea infestation and energy requirements of rodent hosts: are there general rules? | Gete B. & Leykun B. | April 8, 2024 | Title & abstract |
|  | Flea infestation and its control. | Gete B. & Leykun B. | July 3, 2024 | Title & abstract |
|  | Flea infestation as a cause of papular urticaria | Gete B. & Leykun B. | July 3, 2024 | Title & abstract |
|  | Flea infestations: epidemiology, treatment and control | Gete B. & Leykun B. | June 25, 2024 | Title & abstract |
|  | Flea parasitism and host survival in a plague-relevant system: theoretical and conservation implications. | Gete B. & Leykun B. | April 8, 2024 | Title & abstract |
|  | Flea parasitism in wild mammals in the metropolitan region of Sorocaba, São Paulo, Brazil | Gete B. & Leykun B. | May 20, 2024 | Title & abstract |
|  | Fleas (Insecta: Siphonaptera) collected from some small mammals (Mammalia: Rodentia, Eulipotyphla) in Turkey, with new records and new host associations | Gete B. & Leykun B. | May 20, 2024 | Title & abstract |
|  | Fleas and diseases | Gete B. & Leykun B. | April 8, 2024 | Title & abstract |
|  | Fleas and flea-borne diseases | Gete B. & Leykun B. | April 5, 2024 | Title & abstract |
|  | Fleas and flea-borne diseases: biology, control & compliance | Gete B. & Leykun B. | April 5, 2024 | Title & abstract |
|  | Fleas and lesions in armadillo osteoderms | Gete B. & Leykun B. | April 8, 2024 | Title & abstract |
|  | Fleas and ticks in armadillos from Argentinean Patagonia: Diversity, abundance and distribution | Gete B. & Leykun B. | July 3, 2024 | Title & abstract |
|  | Fleas infestation on livestock and stockmen in Mansehra, Northern Pakistan. | Gete B. & Leykun B. | March 18, 2024 | Title & abstract |
|  | Fleas infesting pets in the era of emerging extra-intestinal nematodes | Gete B. & Leykun B. | April 8, 2024 | Title & abstract |
|  | Food-and vector-borne parasitic zoonoses: global burden and impacts | Gete B. & Leykun B. | July 3, 2024 | Title & abstract |
|  | Foot pain in children and adolescents: a problem-based approach in musculoskeletal ultrasonography | Gete B. & Leykun B. | March 18, 2024 | Title & abstract |
|  | Foot pain in the child and adolescent | Gete B. & Leykun B. | April 8, 2024 | Title & abstract |
|  | Foot posture index and body composition measures in children with and without developmental coordination disorder | Gete B. & Leykun B. | July 3, 2024 | Title & abstract |
|  | Foot problems and effectiveness of foot care education in children and adolescents with diabetes mellitus | Gete B. & Leykun B. | April 8, 2024 | Title & abstract |
|  | Foot problems in children presented to the family physician: a comparison between 1987 and 2001 | Gete B. & Leykun B. | April 8, 2024 | Title & abstract |
|  | Four imported cases of tungiasis in Mallorca. | Gete B. & Leykun B. | April 8, 2024 | Title & abstract |
|  | Frequency of childhood dermatological diseases: An analysis of 8551 cases | Gete B. & Leykun B. | April 8, 2024 | Title & abstract |
|  | Fungal infections, infestations, and parasitic infections in neonates and infants | Gete B. & Leykun B. | June 5, 2024 | Title & abstract |
|  | Furuncular myiasis in a child: a case report and literature review | Gete B. & Leykun B. | June 5, 2024 | Title & abstract |
|  | Generalized tungiasis treated with ivermectin | Gete B. & Leykun B. | June 15, 2024 | Title & abstract |
|  | Generalized tungiasis treated with thiabendazole | Gete B. & Leykun B. | April 8, 2024 | Title & abstract |
|  | Genetic variability of Tunga penetrans (Siphonaptera, Tungidae) sand fleas across South America and Africa | Gete B. & Leykun B. | April 8, 2024 | Title & abstract |
|  | Genital Infection by Chigoe Flea in a Pediatric Patient: A Case Report. | Gete B. & Leykun B. | April 8, 2024 | Title & abstract |
|  | Genus Tunga (Siphonaptera: Tungidae) II--neosomes, morphology, classification, and other taxonomic notes. | Gete B. & Leykun B. | March 15, 2024 | Title & abstract |
|  | Geospatial Analysis of Tungiasis Disease Transmission Risk in East Africa | Gete B. & Leykun B. | April 8, 2024 | Title & abstract |
|  | Getting rid of the plague": jiggers removal program in Bungoma, Kenya: Community and health workers perspectives on tungiasis in a high prevalence area. | Gete B. & Leykun B. | March 15, 2024 | Title & abstract |
|  | Global burden of fungal skin diseases: An update from the Global Burden of Diseases Study 2019 | Gete B. & Leykun B. | March 15, 2024 | Title & abstract |
|  | Global burden of skin disease as reflected in Cochrane Database of Systematic Reviews | Gete B. & Leykun B. | April 8, 2024 | Title & abstract |
|  | Global burden of skin disease: inequities and innovations | Gete B. & Leykun B. | May 5, 2024 | Title & abstract |
|  | Global climate change impacts on vector ecology and vector-borne diseases | Gete B. & Leykun B. | May 5, 2024 | Title & abstract |
|  | Global output of research on epidermal parasitic skin diseases from 1967 to 2017 | Gete B. & Leykun B. | May 5, 2024 | Title & abstract |
|  | Global report on neglected tropical diseases 2023 | Gete B. & Leykun B. | April 8, 2024 | Title & abstract |
|  | Got the Travel Bug? A Review of Common Infections, Infestations, Bites, and Stings Among Returning Travelers | Gete B. & Leykun B. | June 5, 2024 | Title & abstract |
|  | Habitat dependence of a parasite-host relationship: flea (Siphonaptera) assemblages in two gerbil species of the Negev Desert | Gete B. & Leykun B. | June 5, 2024 | Title & abstract |
|  | Head lice in hair samples from youths, adults and the elderly in Manaus, Amazonas State, Brazil | Gete B. & Leykun B. | June 5, 2024 | Title & abstract |
|  | Head lice in school children in Uberlandia, Minas Gerais state, Brazil | Gete B. & Leykun B. | April 8, 2024 | Title & abstract |
|  | Head lice infestation in schoolchildren, in Poland—Is there a chance for change? | Gete B. & Leykun B. | April 15, 2024 | Title & abstract |
|  | Head lice infestations in rural Honduras: the need for an integrated approach to control neglected tropical diseases | Gete B. & Leykun B. | April 15, 2024 | Title & abstract |
|  | Head lice were also affected by COVID-19: a decrease on Pediculosis infestation during lockdown in Buenos Aires | Gete B. & Leykun B. | April 15, 2024 | Title & abstract |
|  | Head louse infestations: the “no nit” policy and its consequences | Gete B. & Leykun B. | April 8, 2024 | Title & abstract |
|  | Head pediculosis among in Baghdad area elementary schoolchildren | Gete B. & Leykun B. | April 5, 2024 | Title & abstract |
|  | Health aspects of resettlement in Ethiopia | Gete B. & Leykun B. | April 5, 2024 | Title & abstract |
|  | Health hazards associated with traditional treatment of tungiasis | Gete B. & Leykun B. | April 8, 2024 | Title & abstract |
|  | Healthcare Policies to Eliminate Neglected Tropical Diseases (NTDs) in India: A Roadmap | Gete B. & Leykun B. | April 5, 2024 | Title & abstract |
|  | Helminthic Infections | Gete B. & Leykun B. | June 10, 2024 | Title & abstract |
|  | Hematological parameters of cattle blood in parasitic dermatitis | Gete B. & Leykun B. | April 8, 2024 | Title & abstract |
|  | Hierarchical bayesian logistic modelling of the prevalence and the determinants of tunga penetrans infestation in central, kenya | Gete B. & Leykun B. | June 10, 2024 | Title & abstract |
|  | High exposure to Tunga penetrans (Linnaeus, 1758) correlates with intensity of infestation | Gete B. & Leykun B. | June 10, 2024 | Title & abstract |
|  | High infection rate of Wolbachia endobacteria in the sand flea Tunga penetrans from Brazil | Gete B. & Leykun B. | June 10, 2024 | Title & abstract |
|  | High intensity of Tunga penetrans infection causing severe disease among pigs in Busoga, South Eastern Uganda | Gete B. & Leykun B. | April 8, 2024 | Title & abstract |
|  | High Level of Knowledge about Tungiasis but Little Translation into Control Practices in Karamoja, Northeastern Uganda. | Gete B. & Leykun B. | April 8, 2024 | Title & abstract |
|  | High prevalence of intestinal infections and ectoparasites in dogs, Minas Gerais State (southeast Brazil) | Gete B. & Leykun B. | April 5, 2024 | Title & abstract |
|  | High prevalence of tungiasis in a poor neighbourhood in Fortaleza, Northeast Brazil | Gete B. & Leykun B. | April 8, 2024 | Title & abstract |
|  | High Prevalence Of Tungiasis In A Shantytown In Fortaleza, Northeast Brazil | Gete B. & Leykun B. | April 5, 2024 | Title & abstract |
|  | High prevalence, parasite load, and morbidity in a rural community in Lagos State, Nigeria | Gete B. & Leykun B. | April 8, 2024 | Title & abstract |
|  | Higher proteotoxic stress rather than mitochondrial damage is involved in higher neurotoxicity of bortezomib compared to carfilzomib | Gete B. & Leykun B. | April 8, 2024 | Title & abstract |
|  | High-resolution infrared thermography: a new tool to assess tungiasis-associated inflammation of the skin | Gete B. & Leykun B. | June 10, 2024 | Title & abstract |
|  | Histological Demonstration of the Organisms Causing Human Tungiasis in Eastern Uganda | Gete B. & Leykun B. | April 8, 2024 | Title & abstract |
|  | Histopathological features of tungiasis in Peru | Gete B. & Leykun B. | June 10, 2024 | Title & abstract |
|  | Histopathology of Infectious Diseases: a Practical Approach to Slide Examination and Interpretation: Part II of II | Gete B. & Leykun B. | June 10, 2024 | Title & abstract |
|  | Host body mass, not sex, affects ectoparasite loads in yellow-necked mouse Apodemus flavicollis | Gete B. & Leykun B. | April 8, 2024 | Title & abstract |
|  | Host characteristics and environmental factors differentially drive the burden and pathogenicity of an ectoparasite: a multilevel causal analysis | Gete B. & Leykun B. | July 3, 2024 | Title & abstract |
|  | Host sex, size, and hemoparasite infection influence the effects of ectoparasitic burdens on free‐ranging iguanas | Gete B. & Leykun B. | July 3, 2024 | Title & abstract |
|  | Host-ectoparasite relationships | Gete B. & Leykun B. | July 3, 2024 | Title & abstract |
|  | Host–parasite interactions of rodent hosts and ectoparasite communities from different habitats in Germany | Gete B. & Leykun B. | March 20, 2024 | Title & abstract |
|  | Household food security, morbidity and nutrition status of adult females with tungiasis in murang’a county, kenya | Gete B. & Leykun B. | April 8, 2024 | Title & abstract |
|  | Household Level Factors Associated with Practices towards Water, Sanitation and Hygiene with Occurrence of Diarrhea and Tungiasis among Pupils in Schools with a Feeding Programme in Ganze Sub County, Kenya | Gete B. & Leykun B. | April 8, 2024 | Outcome |
|  | Household-related risk factors of tungiasis and severe disease in Kilifi County, Kenya | Gete B. & Leykun B. | March 10, 2024 | Outcome |
|  | How Do I Differentiate and Treat Bug Bite Reactions? | Gete B. & Leykun B. | June 10, 2024 | Title & abstract |
|  | Human dermatitis caused by Ophionyssus natricis, a snake mite | Gete B. & Leykun B. | June 10, 2024 | Title & abstract |
|  | Human dipylidiasis (dog tapeworm infection) in the United States | Gete B. & Leykun B. | July 3, 2024 | Title & abstract |
|  | Human dipylidiasis: A case report of Dipylidium caninum infection in teaching hospital at Karimnagar | Gete B. & Leykun B. | July 3, 2024 | Title & abstract |
|  | Human immune globulin treatment controls Zika viremia in pregnant rhesus macaques. | Gete B. & Leykun B. | March 20, 2024 | Title & abstract |
|  | Human Infestation by Cat Fleas, Ctenocephalides Felis (Siphonaptera: Pulicidae), from Suburban Raccoons | Gete B. & Leykun B. | April 8, 2024 | Title & abstract |
|  | Human Lice, Bed Bugs, Sand Fleas, Myiasis, and Leeches | Gete B. & Leykun B. | March 10, 2024 | Title & abstract |
|  | Human Tungiasis (Tunga Penetrans) A Report On Three Japanese Infected In Venezuela | Gete B. & Leykun B. | March 10, 2024 | Title & abstract |
|  | Hygiene and skin infections among children below 15 years in Rukindo Primary School, Bushenyi-Ishaka Municipality, Bushenyi District-Western Uganda | Gete B. & Leykun B. | June 10, 2024 | Included |
|  | Hyperpigmented plaque on the foot of a Kenyan patient | Gete B. & Leykun B. | April 8, 2024 | Title & abstract |
|  | Hypertrophy of the organs in the female of Tunga penetrans | Gete B. & Leykun B. | April 10, 2024 | Title & abstract |
|  | Identification of tungiasis infection hotspots with a low-cost, high-throughput method for extracting Tunga penetrans (Siphonaptera) off-host stages from soil samples-An observational study. | Gete B. & Leykun B. | April 8, 2024 | Title & abstract |
|  | Identifying risk factors for tungiasis and heavy infestation in a resource-poor community in northeast Brazil | Gete B. & Leykun B. | June 20, 2024 | Title & abstract |
|  | Illness in travelers returned from Brazil: the GeoSentinel experience and implications for the 2014 FIFA World Cup and the 2016 Summer Olympics | Gete B. & Leykun B. | April 8, 2024 | Title & abstract |
|  | Image Gallery: Tungiasis in four steps. | Gete B. & Leykun B. | July 3, 2024 | Title & abstract |
|  | Images in clinical medicine. Periungual tungiasis | Gete B. & Leykun B. | July 3, 2024 | Title & abstract |
|  | Imaging In Tropical Dermatology | Gete B. & Leykun B. | June 20, 2024 | Title & abstract |
|  | Immune response to fleas in a wild desert rodent: effect of parasite species, parasite burden, sex of host and host parasitological experience | Gete B. & Leykun B. | April 8, 2024 | Title & abstract |
|  | Immune responses to fleas in two rodent species differing in natural prevalence of infestation and diversity of flea assemblages | Gete B. & Leykun B. | April 20, 2024 | Title & abstract |
|  | Immuno-competence and flea parasitism of a desert rodent | Gete B. & Leykun B. | April 8, 2024 | Title & abstract |
|  | Impact of common skin diseases on children in rural Côte d'Ivoire with leprosy and Buruli ulcer co-endemicity: a mixed methods study | Gete B. & Leykun B. | April 8, 2024 | Title & abstract |
|  | Impact of ivermectin administered for scabies treatment on the prevalence of head lice in Atoifi, Solomon Islands | Gete B. & Leykun B. | April 8, 2024 | Title & abstract |
|  | Impact of pediatric dermatologic conditions on child and parent quality of life | Gete B. & Leykun B. | March 20, 2024 | Title & abstract |
|  | Impact of Tungiasis on acquisition of basic education among children aged 5-14 years in Murang’a County, Kenya | Gete B. & Leykun B. | March 20, 2024 | Included |
|  | Impact of Tungiasis on school age children in Muranga county, Kenya | Gete B. & Leykun B. | April 8, 2024 | Title & abstract |
|  | Implementation of the rhesus prevention programme: a prospective study | Gete B. & Leykun B. | March 20, 2024 | Title & abstract |
|  | Implications of Psychosocial Outcomes of Tungiasis Stigma on Health Seeking Behavior among Resource Poor Communities in Central Kenya | Gete B. & Leykun B. | April 8, 2024 | Title & abstract |
|  | Imported and locally acquired human myiasis in Canada: a report of two cases | Gete B. & Leykun B. | June 25, 2024 | Title & abstract |
|  | Imported case of human tungiasis in Hungary | Gete B. & Leykun B. | March 15, 2024 | Title & abstract |
|  | Imported cases of Tungiasis in Mallorca | Gete B. & Leykun B. | March 15, 2024 | Title & abstract |
|  | Imported human tungiasis in Greece | Gete B. & Leykun B. | March 20, 2024 | Title & abstract |
|  | Imported tungiasis | Gete B. & Leykun B. | April 10, 2024 | Title & abstract |
|  | Imported tungiasis in a Finnish journalist: the first case reported from the Nordic countries | Gete B. & Leykun B. | April 10, 2024 | Title & abstract |
|  | Imported tungiasis in a Japanese student returning from East Africa | Gete B. & Leykun B. | April 10, 2024 | Title & abstract |
|  | Imported Tungiasis in a Non-endemic Country | Gete B. & Leykun B. | April 10, 2024 | Title & abstract |
|  | Imported tungiasis: a report of 19 cases and review of the literature. | Gete B. & Leykun B. | April 10, 2024 | Title & abstract |
|  | Imported tungiasis: case report | Gete B. & Leykun B. | June 10, 2024 | Title & abstract |
|  | Incidence and Related Factors of Different Types of Skin Diseases among Children | Gete B. & Leykun B. | April 8, 2024 | Title & abstract |
|  | Incorrectly fitted footwear, foot pain and foot disorders: a systematic search and narrative review of the literature | Gete B. & Leykun B. | April 8, 2024 | Title & abstract |
|  | Increasing incidence of skin disorders in children? A comparison between 1987 and 2001 | Gete B. & Leykun B. | March 20, 2024 | Title & abstract |
|  | Indirect consequences of parental care: sex differences in ectoparasite burden and cleaner-seeking activity in longfin damselfish | Gete B. & Leykun B. | March 10, 2024 | Title & abstract |
|  | Infant and child mortality in Latin America | Gete B. & Leykun B. | April 8, 2024 | Title & abstract |
|  | Infection of the cat flea, Ctenocephalides felis (Bouche) by Neoaplectana carpocapsae Weiser | Gete B. & Leykun B. | March 20, 2024 | Title & abstract |
|  | Infection with the Sand Flea Tunga penetrans (Tungiasis) in a Traveller Returning from Cameroon, Africa | Gete B. & Leykun B. | March 10, 2024 | Title & abstract |
|  | Infection with tungiasis through interhost movement of adult female sand fleas, Tunga penetrans. | Gete B. & Leykun B. | April 15, 2024 | Title & abstract |
|  | Infestation by Tunga penetrans | Gete B. & Leykun B. | April 15, 2024 | Title & abstract |
|  | Infestation of a pediculated cutaneous fibroma by the sand flea. | Gete B. & Leykun B. | April 15, 2024 | Title & abstract |
|  | Infestation of Tunga penetrans in villages near Zomba Central Hospital | Gete B. & Leykun B. | June 20, 2024 | Outcome |
|  | Infestation of Wistar rats with Tunga penetrans in different microenvironments | Gete B. & Leykun B. | June 20, 2024 | Title & abstract |
|  | Infestations in the pediatric patient | Gete B. & Leykun B. | June 20, 2024 | Title & abstract |
|  | Infestations, bites, and insect repellents | Gete B. & Leykun B. | June 20, 2024 | Title & abstract |
|  | Influence of Jigger Infestation on Attendance Rates of Students in Public Day Secondary Schools of Bumula Sub-County, Bungoma County, Keny. | Gete B. & Leykun B. | April 8, 2024 | Outcome |
|  | Insect and arachnid stings, bites, infestations, and repellents | Gete B. & Leykun B. | March 20, 2024 | Title & abstract |
|  | Insect repellents and associated personal protection for a reduction in human disease | Gete B. & Leykun B. | April 8, 2024 | Title & abstract |
|  | Integrated control and management of neglected tropical skin diseases | Gete B. & Leykun B. | March 10, 2024 | Title & abstract |
|  | Integrated management of skin NTDs—lessons learned from existing practice and field research | Gete B. & Leykun B. | April 10, 2024 | Title & abstract |
|  | Integration and scale-up of a primary healthcare-based chronic wound care package for persons affected by skin-NTDs and other conditions in Ethiopia: a protocol for an implementation research study | Gete B. & Leykun B. | April 8, 2024 | Title & abstract |
|  | Integration of management strategies for skin-related neglected tropical diseases | Gete B. & Leykun B. | April 10, 2024 | Title & abstract |
|  | Intensity and clinical morbidities of tungiasis in an impoverished south-west Nigerian community | Gete B. & Leykun B. , Belay D. | April 18, 2024 | Low quality |
|  | Interaction of ectoparasites and their hosts | Gete B. & Leykun B. | April 10, 2024 | Title & abstract |
|  | Intestinal parasitic infections and atopic dermatitis among Venezuelan Warao Amerindian pre‐school children | Gete B. & Leykun B. | April 8, 2024 | Title & abstract |
|  | Investigation of Ctenocephalides felis on domestic dogs and Rickettsia felis infection in the Democratic Republic of Sao Tome and Principe. | Gete B. & Leykun B. | March 16, 2024 | Title & abstract |
|  | Investigation of human tungiasis cases, Sheema District, Uganda, November 2021-February 2022 | Gete B. & Leykun B. | March15, 2024 | Outcome |
|  | Investigations on the biology, epidemiology, pathology and control of Tunga penetrans in Brazil. VI. Natural history of the infestation in laboratory-raised Wistar rats | Gete B. & Leykun B. | April 10, 2024 | Title & abstract |
|  | Investigations on the biology, epidemiology, pathology and control of Tunga penetrans in Brazil: I. Natural history of tungiasis in man | Gete B. & Leykun B. | April 8, 2024 | Title & abstract |
|  | Investigations on the biology, epidemiology, pathology and control of Tunga penetrans in Brazil: III. Cytokine levels in peripheral blood of infected humans | Gete B. & Leykun B. | March 15, 2024 | Title & abstract |
|  | Investigations on the biology, epidemiology, pathology and control of Tunga penetrans in Brazil: IV. Clinical and histopathology | Gete B. & Leykun B. | March 15, 2024 | Title & abstract |
|  | Investigations on the biology, epidemiology, pathology and control of Tunga penetrans in Brazil. V. Cytokine concentrations in experimentally infected Wistar rats | Gete B. & Leykun B. | April 8, 2024 | Title & abstract |
|  | Investigations on the biology, epidemiology, pathology and control of Tunga penetrans in Brazil. II. Prevalence, parasite load and topographic distribution of lesions in the population of a traditional fishing village | Gete B. & Leykun B. | April 8, 2024 | Title & abstract |
|  | Investigations on the biology, epidemiology, pathology, and control of Tunga penetrans in Brazil: VII. The importance of animal reservoirs for human infestation | Gete B. & Leykun B. | June 19, 2024 | Title & abstract |
|  | Investigations on the life cycle and morphology of Tunga penetrans in Brazil | Gete B. & Leykun B. | June 19, 2024 | Title & abstract |
|  | Is a Two Component-Dimeticone Cost effective over the standard treatment: A Markov Modelling of Tungiasis Treatment in Sub-Saharan Africa | Gete B. & Leykun B. | June 19, 2024 | Title & abstract |
|  | Isoxazolines: a novel chemotype highly effective on ectoparasites | Gete B. & Leykun B. | June 19, 2024 | Title & abstract |
|  | Jigger flea infestation (tungiasis) in rural western Tanzania: high prevalence and severe morbidity | Gete B. & Leykun B. | June 19, 2024 | Included |
|  | Jigger infestation a menace to children's school attendence. | Gete B. & Leykun B. | June 19, 2024 | Title & abstract |
|  | Jigger infestation in children in a rural area of Rivers State of Nigeria. | Gete B. & Leykun B. | June 19, 2024 | Title & abstract |
|  | Jigger infestation in poor resource communities in Africa Consultancy African Intelligence. | Gete B. & Leykun B. | June 19, 2024 | Title & abstract |
|  | Jigger persistence and associated factors among households in Mayuge District, Uganda | Gete B. & Leykun B. | June 19, 2024 | Title & abstract |
|  | Jigger-Control Related Health Issues and Suggested Interventions in Iganga District, Eastern Uganda | Gete B. & Leykun B. | June 19, 2024 | Outcome |
|  | Jiggers Detrimental To Pupils' Outdoor Play Activities | Gete B. & Leykun B. | June 19, 2024 | Outcome |
|  | Jiggers outbreak in Uganda | Gete B. & Leykun B. | June 19, 2024 | Title & abstract |
|  | Knowledge, Attitude and Practices of Individuals Aged Between 10 to 45 Years on Jigger Infestation in Namalu Sub-county Nakapiripirit District, Uganda | Gete B. & Leykun B. | June 19, 2024 | Title & abstract |
|  | Knowledge, attitude and practices on jigger infestation among household members aged 18 to 60 years: case study of a rural location in Kenya | Gete B. & Leykun B. | June 19, 2024 | Title & abstract |
|  | Knowledge, Attitude And Practices Regarding Tungiasis In Population Of Lahore, Punjab, Pakistan. | Gete B. & Leykun B. | June 19, 2024 | Title & abstract |
|  | Knowledge, Attitude, and Practice of Rural Households in Bojnourd, Iran Regarding the Fleas | Gete B. & Leykun B. | June 19, 2024 | Title & abstract |
|  | Knowledge, attitudes, perceptions, and practices regarding cutaneous larva migrans in deprived communities in Manaus, Brazil | Gete B. & Leykun B. | June 19, 2024 | Title & abstract |
|  | Knowledge, perception and attitudes of Zilindo community towards prevalence of Tungiasis | Gete B. & Leykun B. | June 15, 2024 | Title & abstract |
|  | Lesions in osteoderms of pampatheres (Mammalia, Xenarthra, Cingulata) possibly caused by fleas | Gete B. & Leykun B. | June 15, 2024 | Title & abstract |
|  | Lesions of the lid margins caused by ectoparasites in India | Gete B. & Leykun B. | June 15, 2024 | Title & abstract |
|  | Leukocytosis and blood eosinophilia in a polyparasitised population in north-eastern Brazil | Gete B. & Leykun B. | June 16, 2024 | Title & abstract |
|  | Life threat and posttraumatic stress in school-age children | Gete B. & Leykun B. | June 16, 2024 | Title & abstract |
|  | Limitations and opportunities of smallholders' practical knowledge when dealing with pig health issues in northern Uganda | Gete B. & Leykun B. | June 15, 2024 | Title & abstract |
|  | Limping in children: look what the cat dragged in! | Gete B. & Leykun B. | April 16, 2024 | Title & abstract |
|  | Lotilaner-a novel systemic tick and flea control product for dogs | Gete B. & Leykun B. | June 10, 2024 | Title & abstract |
|  | Magnitude and associated risk factors of superficial skin fungal infection among primary school children in Southern Tanzania | Gete B. & Leykun B. | June 10, 2024 | Title & abstract |
|  | Magnitude of flat foot and its associated factors among school-aged children in Southern Ethiopia: an institution-based cross-sectional study | Gete B. & Leykun B. | June 8, 2024 | Title & abstract |
|  | Major health problems in some selected districts of Tanzania | Gete B. & Leykun B. | June 19, 2024 | Title & abstract |
|  | Management of very severe tungiasis cases through repeated community-based treatment with a dimeticone oil formula: A longitudinal study in a hyperendemic region in Uganda | Gete B. & Leykun B. | June 19, 2024 | Title & abstract |
|  | Managing communication surrounding tungiasis stigma in Kenya | Gete B. & Leykun B. | June 19, 2024 | Title & abstract |
|  | Manifestations and management of flea-borne rickettsioses | Gete B. & Leykun B. | June 19, 2024 | Title & abstract |
|  | Mapping the geographic distribution of tungiasis in Sub-Saharan Africa | Gete B. & Leykun B. | June 19, 2024 | Title & abstract |
|  | Material on the Ecology of Three Species of Great Sand Rat Fleas from a Comparative Aspect A | Gete B. & Leykun B. | June 19, 2024 | Title & abstract |
|  | Mathematical Model for the Effects of Intervention Measures on the Transmission Dynamics of Tungiasis | Gete B. & Leykun B. | June 19, 2024 | Title & abstract |
|  | Mathematical model on the impact of protection against tungiasis transmission dynamics | Gete B. & Leykun B. | June 19, 2024 | Title & abstract |
|  | Mathematical modeling of the effects of public health education on tungiasis—a neglected disease with many challenges in endemic communities | Gete B. & Leykun B. | June 19, 2024 | Title & abstract |
|  | Mathematical modeling of the effects of public health education on tungiasis | Gete B. & Leykun B. | June 19, 2024 | Title & abstract |
|  | Mathematical modelling of tungiasis disease dynamics incorporating hygiene as a control strategy | Gete B. & Leykun B. | May 15,2024 | Title & abstract |
|  | Measuring educational neglect using the Q method: A model based on the burden of disseminated tungiasis | Gete B. & Leykun B. | May 15, 2024 | Title & abstract |
|  | Medical and veterinary entomology. Bologna, Italy | Gete B. & Leykun B. | May 15, 2024 | Title & abstract |
|  | Meningeal Signs and Cerebellar Involvement in Scrub Typhus: A Case Report | Gete B. & Leykun B. | May 15, 2024 | Title & abstract |
|  | Mental health, stigma, and neglected tropical diseases: A review and systematic mapping of the evidence | Gete B. & Leykun B. | June 12, 2024 | Title & abstract |
|  | Mite‐transmitted dermatoses and infectious diseases in returning travelers | Gete B. & Leykun B. | June 19, 2024 | Title & abstract |
|  | Modeling heat transfer in tungiasis inflammation of the skin. | Gete B. & Leykun B. | June 19, 2024 | Title & abstract |
|  | Modeling the impact of public health education on tungiasis dynamics with saturated treatment: Insight through the Caputo fractional derivative | Gete B. & Leykun B. | June 19, 2024 | Title & abstract |
|  | Modelling Jiggers Infestation And Interventions In Humans: A Case Study Of Murang'a County, Kenya | Gete B. & Leykun B. | June 19, 2024 | Title & abstract |
|  | Modelling prevention and control of jigger infestation in Mayuge district: a mathematical approach. | Gete B. & Leykun B. | June 19, 2024 | Title & abstract |
|  | Modelling the dynamics of jiggers infestation: insights from a theoretical model | Gete B. & Leykun B. | June 19, 2024 | Title & abstract |
|  | Modelling the dynamics of Tungiasis transmission in zoonotic areas | Gete B. & Leykun B. | June 19, 2024 | Title & abstract |
|  | Molecular biological investigations of Brazilian Tunga sp. isolates from man, dogs, cats, pigs and rats | Gete B. & Leykun B. | June 19, 2024 | Title & abstract |
|  | Molecular characterization of Tunga trimamillata and T. penetrans (Insecta, Siphonaptera, Tungidae): taxonomy and genetic variability | Gete B. & Leykun B. | June 19, 2024 | Title & abstract |
|  | Molecular Evidence of Perinatal Transmission of Bartonella vinsonii subsp. berkhoffii and Bartonella henselae to a Child | Gete B. & Leykun B. | June 19, 2024 | Title & abstract |
|  | Morbidity assessment in sand flea disease (tungiasis) | Gete B. & Leykun B. | June 19, 2024 | Title & abstract |
|  | Morbidity, Risk Factors, and flea species responsible for Tungiasis in selected villages in Kisumu County, Kenya | Gete B. & Leykun B. | June 15, 2024 | Included |
|  | Morsus insecti from flea bites: seasonal appearance and relation to sex and age of flea‐exposed persons | Gete B. & Leykun B. | June 10, 2024 | Title & abstract |
|  | Multi-objective optimal control of Tungiasis diseases with terminal demands | Gete B. & Leykun B. | June 15,2024 | Title & abstract |
|  | Multiple infestation with Dipylidium caninum in an infant | Gete B. & Leykun B. | June 15, 2024 | Title & abstract |
|  | Multiple juckende Papeln mit zentralen schwarzen Punkten an den Extremitäten einer Landarbeiterin | Gete B. & Leykun B. | June 15, 2024 | Title & abstract |
|  | Multiple pruritic papules with central black dots on the extremities of a farmworker | Gete B. & Leykun B. | June 15, 2024 | Title & abstract |
|  | Multiple, black, burning papules on the soles of the feet of a 20-year-old woman | Gete B. & Leykun B. | June 15, 2024 | Title & abstract |
|  | Murine typhus outbreak presenting as multisystem inflammatory syndrome in children during SARS-CoV-2 pandemic | Gete B. & Leykun B. | June 15,  2024 | Title & abstract |
|  | Myiasis (Muscoidea, Oestroidea) | Gete B. & Leykun B. | June 15, 2024 | Title & abstract |
|  | Myiasis and tungiasis | Gete B. & Leykun B. | June 5,  2024 | Title & abstract |
|  | Myiasis in a scalp wound as a complication of neglected pediculosis capitis: a case report. | Gete B. & Leykun B. | June 5, 2024 | Title & abstract |
|  | Myth or truth: investigation of the jumping ability of Tunga penetrans (Siphonaptera: Tungidae) | Gete B. & Leykun B. | June 5, 2024 | Title & abstract |
|  | Nasal septal ulceration and perforation in jiggers | Gete B. & Leykun B. | June 5, 2024 | Title & abstract |
|  | National policy guidelines on prevention and control of jigger’s infestation. Division of Environmental Health, Ministry of Health, Kenya | Gete B. & Leykun B. | June 5, 2024 | Title & abstract |
|  | National prevalence and risk factors for tungiasis in Kenya | Gete B. & Leykun B. | June 5,  2024 | Outcome |
|  | Negative effect of ectoparasite burdens on the condition factor from farmed tilapia Oreochromis niloticus in the Yucatan, Mexico | Gete B. & Leykun B. | June 5, 2024 | Title & abstract |
|  | Neglected diseases of neglected populations: thinking to reshape the determinants of health in Latin America and the Caribbean. | Gete B. & Leykun B. | March 21, 2024 | Title & abstract |
|  | Neglected tropical diseases: epidemiology and global burden | Gete B. & Leykun B. | March 21, 2024 | Title & abstract |
|  | Neosomes of tungid fleas on wild and domestic animals | Gete B. & Leykun B. | March 21, 2024 | Title & abstract |
|  | Nest-type associated microclimatic conditions as potential drivers of ectoparasite infestations in African penguin nests | Gete B. & Leykun B. | March 10, 2024 | Title & abstract |
|  | Neurocognitive and mental health outcomes in children with tungiasis: a cross-sectional study in rural Kenya and Uganda | Gete B. & Leykun B. | March 10, 2024 | Outcome |
|  | New distribution and host records for Hectopsylla pulex Haller (Siphonaptera, Tungidae) with notes on biology and morphology | Gete B. & Leykun B. | March10, 2024 | Title & abstract |
|  | New records of human tungiasis in Mexico (Siphonaptera: Tungidae) | Gete B. & Leykun B. | April 15, 2024 | Title & abstract |
|  | Nine cases of human dipylidiasis in Moscow region during 1987 to 2017. | Gete B. & Leykun B. | April 15, 2024 | Title & abstract |
|  | Nitric Peroxide as a Disinfectant in Plague and Jigger | Gete B. & Leykun B. | April 15, 2024 | Title & abstract |
|  | Not a simple plantar wart: a case of tungiasis | Gete B. & Leykun B. | April 15, 2024 | Title & abstract |
|  | Observations on the endemicity of plague in Karatu and Ngorongoro, northern Tanzania | Gete B. & Leykun B. | June 20, 2024 | Title & abstract |
|  | Occurrence and impact of zoonoses in pet dogs and cats at US Air Force bases. | Gete B. & Leykun B. | June 20, 2024 | Title & abstract |
|  | Occurrence of the off-host life stages of Tunga penetrans (Siphonaptera) in various environments in Brazil | Gete B. & Leykun B. | May 15, 2024 | Title & abstract |
|  | Occurrence of Tungiasis in Ceara State, Northeast Brazil: Results of a Rapid Assessment Method | Gete B. & Leykun B. | May 15, 2024 | Title & abstract |
|  | Occurrence of tungiasis in dogs from São Paulo municipality | Gete B. & Leykun B. | May 15,  2024 | Title & abstract |
|  | Official development assistance for health–how neglected are neglected tropical diseases? An analysis of health financing | Gete B. & Leykun B. | June 5, 2024 | Title & abstract |
|  | On a new species of sand flea from Ecuador and tungiasis, a problem of public health in many developing countries | Gete B. & Leykun B. | June 5,  2024 | Title & abstract |
|  | Onchodermatitis—correlation between skin disease and parasitic load in an endemic focus in Ecuador | Gete B. & Leykun B. | June 5,  2024 | Title & abstract |
|  | One jigger, no complications | Gete B. & Leykun B. | May 15,  2024 | Title & abstract |
|  | Open season: select the best 'beast' for each skin lesion | Gete B. & Leykun B. | May 15,  2024 | Title & abstract |
|  | Opportunistic tropical infections in immunosuppressed patients | Gete B. & Leykun B. | May 15,  2024 | Title & abstract |
|  | Optimal control techniques on a mathematical model for the dynamics of tungiasis in a community | Gete B. & Leykun B. | June 12,  2024 | Title & abstract |
|  | Optimized Deformable Model-based Segmentation and Deep Learning for Lung Cancer Classification | Gete B. & Leykun B. | June 12, 2024 | Title & abstract |
|  | Oral ivermectin for treatment of pediculosis capitis | Gete B. & Leykun B. | June 12,  2024 | Title & abstract |
|  | Outbreak of scabies among preschool children, Accra, Ghana, 2017 | Gete B. & Leykun B. | June 12,  2024 | Title & abstract |
|  | Outbreak of Tungiasis following a trip to Ethiopia | Gete B. & Leykun B. | May10, 2024 | Title & abstract |
|  | Overview of infectious and non-infectious diseases in French Guiana in 2022 | Gete B. & Leykun B. | May 10,  2024 | Title & abstract |
|  | Overweight, obesity and foot posture in children: a cross‐sectional study | Gete B. & Leykun B. | April 15,  2024 | Title & abstract |
|  | Pain, pruritus, and swelling localized to two toes. Tungiasis | Gete B. & Leykun B. | June 20, 2024 | Title & abstract |
|  | Painful nodule on the foot of a traveller returning from Africa | Gete B. & Leykun B. | June 20, 2024 | Title & abstract |
|  | Papular urticaria in children | Gete B. & Leykun B. | April 15, 2024 | Title & abstract |
|  | Parasite burdens and host susceptibility of Zebu and N'Dama cattle in village herds in Gambia | Gete B. & Leykun B. | April 15, 2024 | Title & abstract |
|  | Parasites causing cutaneous wounds: Theory and practice from a dermatological point of view | Gete B. & Leykun B. | April 15, 2024 | Title & abstract |
|  | Parasites of importance for human health in Nigerian dogs: high prevalence and limited knowledge of pet owners | Gete B. & Leykun B. | June 20, 2024 | Title & abstract |
|  | Parasitic Dermatitis Due To Canine Demodicosis In Dogs | Gete B. & Leykun B. | March 12, 2024 | Title & abstract |
|  | Parasitic dermatitis in canines of Jammu region | Gete B. & Leykun B. | March 12, 2024 | Title & abstract |
|  | Parasitic dermatitis of the muzzle of a cow with clinical signs of leukoderma. | Gete B. & Leykun B. | June 20, 2024 | Title & abstract |
|  | Parasitic diseases in travelers: a focus on therapy | Gete B. & Leykun B. | April 15, 2024 | Title & abstract |
|  | Parasitic diseases with cutaneous manifestations | Gete B. & Leykun B. | April 15, 2024 | Title & abstract |
|  | Parasitic Granulomatous Dermatitis Caused by Pelodera spp. in Buffalo on Marajó Island, Pará | Gete B. & Leykun B. | June 20, 2024 | Title & abstract |
|  | Parasitic hypertrophy of female sand fleas (Tunga Penetrans). | Gete B. & Leykun B. | June 20, 2024 | Title & abstract |
|  | Parasitic infections in travelers and immigrants: part II helminths and ectoparasites | Gete B. & Leykun B. | June 20, 2024 | Title & abstract |
|  | Parasitic infections of the skin and subcutaneous tissues | Gete B. & Leykun B. | June 20, 2024 | Title & abstract |
|  | Parasitic otitis and dermatitis in dogs in Tuscany Otiti e dermatiti parassitarie nei cani della Toscana | Gete B. & Leykun B. | June 20, 2024 | Title & abstract |
|  | Parasitic skin diseases: health care‐seeking in a slum in north‐east Brazil | Gete B. & Leykun B. | June 20, 2024 | Title & abstract |
|  | Parasitic skin infections in the elderly: recognition and drug treatment | Gete B. & Leykun B. | July 3, 2024 | Title & abstract |
|  | Pathogens in fleas collected from cats and dogs: distribution and prevalence in the UK. | Gete B. & Leykun B. | July 3, 2024 | Title & abstract |
|  | Pathology and diagnosis of proliferative and ulcerative dermatitis associated with Tunga penetrans infestation in cattle. | Gete B. & Leykun B. | June 20, 2024 | Title & abstract |
|  | Pathology and diagnosis of proliferative dermatitis produced by Tunga penetrans in cattle | Gete B. & Leykun B. | July 3, 2024 | Title & abstract |
|  | Patient education in chronic skin diseases: a systematic review | Gete B. & Leykun B. | July 3, 2024 | Title & abstract |
|  | Pattern of childhood dermatoses at a teaching hospital of Saudi Arabia | Gete B. & Leykun B. | June 20, 2024 | Title & abstract |
|  | Pattern of common skin conditions among school children in an urban area of a district in coastal Karnataka: A cross-sectional study | Gete B. & Leykun B. | March 15, 2024 | Title & abstract |
|  | Pattern of common skin diseases among school going children in Semi-Urban Area of Jaipur: A cross-sectional study | Gete B. & Leykun B. | June 20, 2024 | Title & abstract |
|  | Pattern of dermatoses in preschool children in a teaching hospital in Uttarakhand, India | Gete B. & Leykun B. | March 15, 2024 | Title & abstract |
|  | Pattern of paediatric dermatoses at dermatology clinics in Ile-Ife and Ilesha, Nigeria | Gete B. & Leykun B. | March 15, 2024 | Title & abstract |
|  | Pattern of pediatric dermatoses at a referral centre | Gete B. & Leykun B. | March 15, 2024 | Title & abstract |
|  | Pattern of skin diseases among preschool and primary school-aged children in Dermatology Damietta Hospital | Gete B. & Leykun B. | June 20, 2024 | Title & abstract |
|  | Pattern of skin diseases amongst children attending a dermatology clinic in Lagos, Nigeria | Gete B. & Leykun B. | April 15, 2024 | Title & abstract |
|  | Pattern of skin diseases in children attending a dermatology clinic in a referral hospital in Wolaita Sodo, southern Ethiopia | Gete B. & Leykun B. | June 20, 2024 | Title & abstract |
|  | Pattern of skin diseases in children attending the dermatology clinic in Alert referral hospital, Addis Ababa, Ethiopia: A retrospective study | Gete B. & Leykun B. | March 20, 2024 | Title & abstract |
|  | Pattern of skin diseases in patients visiting a tertiary care health facility at Hyderabad, Pakistan | Gete B. & Leykun B. | March 20, 2024 | Title & abstract |
|  | Pattern of Skin Disorder among School Children in Port-Harcourt, Rivers State | Gete B. & Leykun B. | June 20, 2024 | Title & abstract |
|  | Patterns of coexistence: ectoparasites on small mammals in northern Fennoscandia | Gete B. & Leykun B. | April 15, 2024 | Title & abstract |
|  | Patterns of Common Dermatological Conditions among Children and Adolescents in Pakistan | Gete B. & Leykun B. | April 15, 204 | Title & abstract |
|  | Patterns of skin diseases among pediatric patients attending the pediatric dermatological clinic at King Hussein Medical Center | Gete B. & Leykun B. | June 10, 2024 | Title & abstract |
|  | Pediatric dermatology in practice: spectrum of skin diseases and approach to patients at a Turkish pediatric dermatology center | Gete B. & Leykun B. | June 20, 2024 | Title & abstract |
|  | Pediatric dermatoses encountered in dermatology outpatient department of a teaching institute | Gete B. & Leykun B. | April 15, 2024 | Title & abstract |
|  | Pediatric dermatoses in India | Gete B. & Leykun B. | April 15, 2024 | Title & abstract |
|  | Pediatric foot and ankle disorders | Gete B. & Leykun B. | April 15, 2024 | Title & abstract |
|  | Pediatric obesity and skin disease: cutaneous findings and associated quality-of-life impairments in 103 children and adolescents with obesity | Gete B. & Leykun B. | June 20, 2024 | Title & abstract |
|  | Pediculosis among primary school children in Calabar, Nigeria and implications for control | Gete B. & Leykun B. | June 20, 2024 | Title & abstract |
|  | Pediculosis capitis: prevalence and its associated factors in primary school children living in rural and urban areas in Kayseri, Turkey | Gete B. & Leykun B. | June 20, 2024 | Title & abstract |
|  | Pediculosis humanus capitis: An Emerging Ectoparasitic Infection among Children in Maiduguri Internally Displaced Camps (IDPs), Borno State, Nigeria | Gete B. & Leykun B. | June 20, 2024 | Title & abstract |
|  | Perceptions of Problems with Household Insects: Qualitative and Quantitative Findings from Peri-Urban Communities in Arequipa, Peru | Gete B. & Leykun B. | March 15, 2024 | Title & abstract |
|  | Periungual Tungiasis | Gete B. & Leykun B. | March 15, 2024 | Title & abstract |
|  | Periungual tungiasis in the Democratic Republic of Sao Tome and Principe | Gete B. & Leykun B. | March 15, 2024 | Title & abstract |
|  | Personal hygiene practices and related skin diseases among primary school children of urban poor locality | Gete B. & Leykun B. | June 20, 2024 | Title & abstract |
|  | Pet animal infestations and human skin lesions | Gete B. & Leykun B. | April 10, 2024 | Title & abstract |
|  | Phenology determines seasonal variation in ectoparasite loads in a natural insect population | Gete B. & Leykun B. | April 10, 2024 | Title & abstract |
|  | Phenotype–environment matching in sand fleas | Gete B. & Leykun B. | April 10, 2024 | Title & abstract |
|  | Phlebotomine sand flies and moth flies (Psychodidae) | Gete B. & Leykun B. | April 10, 2024 | Title & abstract |
|  | Photo quiz: risk in travelers after barefoot sand walking in tropical countries. Tunga penetrans | Gete B. & Leykun B. | April 10, 2024 | Title & abstract |
|  | Physical and physiological costs of ectoparasitic mites on host flight endurance | Gete B. & Leykun B. | April 10, 2024 | Title & abstract |
|  | Pigs are the most important animal reservoir for Tunga penetrans (jigger flea) in rural Nigeria. | Gete B. & Leykun B. | June 20, 2024 | Title & abstract |
|  | Podoconiosis in Uganda: prevalence, geographical distribution and risk factors | Gete B. & Leykun B. | July 3, 2024 | Title & abstract |
|  | Point prevalence surveys of antimicrobial use among eight neonatal intensive care units in India: 2016 | Gete B. & Leykun B. | July 3, 2024 | Title & abstract |
|  | Postural Changes In Children And Adolescents With Foot Abnormalities--A Systematic Review. | Gete B. & Leykun B. | June 20, 2024 | Title & abstract |
|  | Potential use of dermoscopy in atypical tungiasis | Gete B. & Leykun B. | March 20, 2024 | Title & abstract |
|  | Preliminary report on infestation by chiggers or Tunga penetrans in the state of Nayarit. | Gete B. & Leykun B. | March 10, 2024 | Title & abstract |
|  | Presence of dogs and proximity to a wildlife reserve increase household level risk of tungiasis in Kwale, Kenya. | Gete B. & Leykun B. | April 20, 2024 | Outcome |
|  | Presence of Tunga trimamillata (Insecta, Siphonaptera) in Peru | Gete B. & Leykun B. | June 20, 2024 | Title & abstract |
|  | Present and future: Infectious tropical travel rashes and the impact of climate change | Gete B. & Leykun B. | June 20, 2024 | Title & abstract |
|  | Prevalence and associated factors of scabies among schoolchildren in Dabat district, northwest Ethiopia, 2018 | Gete B. & Leykun B. | June 20, 2024 | Title & abstract |
|  | Prevalence and associated factors of Tunga penetrans infestation among 5-14-year-olds in rural Ethiopia. | Gete B. & Leykun B. | June 20, 2024 | Included |
|  | Prevalence And Associated Risk Factors of Tungiasis in Chipata and Vubwi Districts of Eastern Zambia. | Gete B. & Leykun B. | April 15, 2024 | Outcome |
|  | Prevalence and clinical aspects of Tungiasis in south-west Nigerian schoolchildren | Gete B. & Leykun B. | April 15, 2024 | Included |
|  | Prevalence and diversity of ectoparasites in scavenging chickens (Gallus domesticus) and their association to body weight | Gete B. & Leykun B. | June 15, 2024 | Title & abstract |
|  | Prevalence and factors associated with intestinal parasitic infection among under-five children in and around Haro Dumal Town, Bale Zone, Ethiopia | Gete B. & Leykun B. | April 20, 2024 | Title & abstract |
|  | Prevalence And Factors Associated With The Management Of Tungiasis In Ikolomani Sub-County, Kakamega County, Western Kenya | Gete B. & Leykun B. | April 15, 2024 | Outcome |
|  | Prevalence and hemato-biochemical studies in parasitic and non-parasitic dermatological disorders in Surti buffalo and buffalo calves. | Gete B. & Leykun B. | April 15, 2024 | Title & abstract |
|  | Prevalence and Infection Intensity of Human and Animal Tungiasis in Napak District, Karamoja, Northeastern Uganda. | Gete B. & Leykun B. | April 20, 2024 | Outcome |
|  | Prevalence and intensity of fleas parasitizing an isolated population of screaming hairy armadillo in Buenos Aires province, Argentina: host-related factors and temporal dynamics | Gete B. & Leykun B. | April 20, 2024 | Title & abstract |
|  | Prevalence and intensity of the ectoparasite Echinophthirius horridus on harbour seals (Phoca vitulina): effects of host age and inter-annual variability in host food | Gete B. & Leykun B. | April 20, 2024 | Title & abstract |
|  | Prevalence and pattern of dermatological disorders in the pediatric emergency service | Gete B. & Leykun B. | April 20, 2024 | Title & abstract |
|  | Prevalence and pattern of skin diseases among school age children at the University of Port Harcourt Teaching Hospital, Nigeria: A hospital based study | Gete B. & Leykun B. | April 20, 2024 | Title & abstract |
|  | Prevalence and pattern of skin disorders among female schoolchildren in Eastern Saudi Arabia | Gete B. & Leykun B. | April 20, 2024 | Title & abstract |
|  | Prevalence and Risk Factors Associated with Tungiasis among School-Aged Children in the City of Port Gentil, Ogooué-Maritime Province, Gabon in Central Africa | Gete B. & Leykun B. | April 15, 2024 | Included |
|  | Prevalence and risk factors associated with tungiasis in Mayuge district, Eastern Uganda. | Gete B. & Leykun B. | April 20, 2024 | Title & abstract |
|  | Prevalence and risk factors for intestinal parasite infections in schoolchildren, in the city of Santarém, Pará State, Brazil | Gete B. & Leykun B. | April 20, 2024 | Title & abstract |
|  | Prevalence and risk factors of hookworm-related cutaneous larva migrans (HrCLM) in a resource-poor community in Manaus, Brazil. | Gete B. & Leykun B. | March 10, 2024 | Title & abstract |
|  | Prevalence and risk factors of tungiasis among children of Wensho district, southern Ethiopia | Gete B. & Leykun B. | April 20, 2024 | Included |
|  | Prevalence and socio-demographic determinants of skin disease among lower primary school children in Calicut, Kerala | Gete B. & Leykun B. | March 10, 2024 | Title & abstract |
|  | Prevalence and Spectrum of Skin Disorders Among Children Attending the General Out-Patient Clinic of Federal Medical Center Lokoja Kogi State | Gete B. & Leykun B. | April 20, 2024 | Title & abstract |
|  | Prevalence and Types of Skin Diseases Seen among Children Attending the Children's Outpatient Clinic in a Tertiary Care Hospital in Southern Nigeria-A Descriptive | Gete B. & Leykun B. | March 10, 2024 | Title & abstract |
|  | Prevalence of atopic dermatitis in Italian schoolchildren: factors affecting its variation | Gete B. & Leykun B. | April 20, 2024 | Title & abstract |
|  | Prevalence of atopic dermatitis, asthma, allergic rhinitis, and hand and contact dermatitis in adolescents. The Odense Adolescence Cohort Study on Atopic Diseases | Gete B. & Leykun B. | March 10, 2024 | Title & abstract |
|  | Prevalence of Canine Parasitic Dermatitis in and Around Jammu | Gete B. & Leykun B. | March 20, 2024 | Title & abstract |
|  | Prevalence of childhood acne, ephelides, warts, atopic dermatitis, psoriasis, alopecia areata and keloid in Kaohsiung County, Taiwan: a community‐based clinical | Gete B. & Leykun B. | April 20, 2024 | Title & abstract |
|  | Prevalence of common dermatoses in school children of rural areas of Salem; a region of South India | Gete B. & Leykun B. | March 20, 2024 | Title & abstract |
|  | Prevalence of Dermatological Disease Among Persons 1-74 Years of Age, United States | Gete B. & Leykun B. | June 10, 2024 | Title & abstract |
|  | Prevalence of dermatological lesions in hospitalized children at the University College Hospital, Ibadan, Nigeria | Gete B. & Leykun B. | April 20, 2024 | Title & abstract |
|  | Prevalence of Dermatoses and Associated Factors Among School Children in Vijayapura, Karnataka | Gete B. & Leykun B. | April 20, 2024 | Title & abstract |
|  | Prevalence of ectoparasites in cattle (Bos indicus) of Jessore, Bangladesh | Gete B. & Leykun B. | March 25, 2024 | Title & abstract |
|  | Prevalence of ectoparasites in free-range backyard chickens, domestic pigeons (Columba livia domestica) and turkeys of Kermanshah province, west of Iran | Gete B. & Leykun B. | March 10, 2024 | Title & abstract |
|  | Prevalence of ectoparasites in goat at Gazipur in Bangladesh | Gete B. & Leykun B. | June 10, 2024 | Title & abstract |
|  | Prevalence of ectoparasites infestation of chicken in three poultry farms in Awka | Gete B. & Leykun B. | June 10, 2024 | Title & abstract |
|  | Prevalence of ectoparasitic infections and other dermatological infections and their associated factors among School children in Gampaha District, Sri Lanka | Gete B. & Leykun B. | April 20, 2024 | Title & abstract |
|  | Prevalence of intestinal parasites among individuals with allergic skin diseases | Gete B. & Leykun B. | April 15, 2024 | Title & abstract |
|  | Prevalence of jigger flea (Tunga Penetrans) infestation amongst age groups, male and female persons in Jinja District, Eastern Uganda | Gete B. & Leykun B., Belay D. | April 20, 2024 | Low quality |
|  | Prevalence of lameness and hoof lesions in all year-round grazing cattle in Brazil | Gete B. & Leykun B. | April 20, 2024 | Title & abstract |
|  | Prevalence of major ectoparasites of calves and associated risk factors in and around Bishoftu town | Gete B. & Leykun B. | April 15, 2024 | Title & abstract |
|  | Prevalence of papular urticaria caused by flea bites and associated factors in children 1–6 years of age in Bogotá, DC | Gete B. & Leykun B. | April 20, 2024 | Title & abstract |
|  | Prevalence of pediatric dermatoses in a university hospital in southeastern Brazil | Gete B. & Leykun B. | March 20, 2024 | Title & abstract |
|  | Prevalence of pediculosis capitis among Korean children | Gete B. & Leykun B. | April 20, 2024 | Title & abstract |
|  | Prevalence of Skin Disease and Its Associated Factors Among Primary Schoolchildren: A Cross-Sectional Study from a Northern Ethiopian Town | Gete B. & Leykun B. | March 10, 2024 | Title & abstract |
|  | Prevalence of skin disease in rural Tanzania and factors influencing the choice of health care, modern or traditional | Gete B. & Leykun B. | March 19, 2024 | Title & abstract |
|  | Prevalence of skin diseases among pediatric patients in Turkey | Gete B. & Leykun B. | April 15, 2024 | Title & abstract |
|  | Prevalence of skin diseases among primary school children | Gete B. & Leykun B. | March 19, 2024 | Title & abstract |
|  | Prevalence of Skin Diseases Among Primary School Children in Benha City, Kalubia Governorate, Egypt | Gete B. & Leykun B. | April 20, 2024 | Title & abstract |
|  | Prevalence of skin diseases and prognosis of atopic dermatitis in primary school children in populated areas of Japan from 2010 to 2019: the Asa Study in Hiroshima | Gete B. & Leykun B. | March 19, 2024 | Title & abstract |
|  | Prevalence of skin diseases in Cameroonian children and adolescents: Insights into outpatient units at Laquintinie, Cameroon. | Gete B. & Leykun B. | June 20, 2024 | Title & abstract |
|  | Prevalence of skin diseases in children 1 to 6 years old in the city of Bogota, Colombia | Gete B. & Leykun B. | June 20, 2024 | Title & abstract |
|  | Prevalence of Skin Diseases in Children Admitted to Mersin University School of Medicine, Dermatology Clinic | Gete B. & Leykun B. | March 19, 2024 | Title & abstract |
|  | Prevalence of skin diseases in children attending government vs private school in a rural set up in the sub-Himalayan region | Gete B. & Leykun B. | June 20, 2024 | Title & abstract |
|  | Prevalence of skin diseases in pre-school children aged 0-7 years in 12 cities of China | Gete B. & Leykun B. | March 19, 2024 | Title & abstract |
|  | Prevalence of skin diseases in school children in rural and urban communities in the Illubabor province, south-western Ethiopia: a preliminary survey | Gete B. & Leykun B. | July 3, 2024 | Title & abstract |
|  | Prevalence of skin diseases in school-age children | Gete B. & Leykun B. | July 3, 2024 | Title & abstract |
|  | Prevalence of skin disorders among male schoolchildren in Amman, Jordan | Gete B. & Leykun B. | March 19, 2024 | Title & abstract |
|  | Prevalence of skin disorders among primary school children in Diyarbakir, Turkey | Gete B. & Leykun B. | April 20, 2024 | Title & abstract |
|  | Prevalence of skin disorders among primary-school children in Baghdad governorate, Iraq | Gete B. & Leykun B. | March 19, 2024 | Title & abstract |
|  | Prevalence of skin disorders in primary and secondary school age children in Canakkale, Turkey: a community-based survey | Gete B. & Leykun B. | April 15, 2024 | Title & abstract |
|  | Prevalence of skin Neglected Tropical Diseases and superficial fungal infections in two peri-urban schools and one rural community setting in Togo | Gete B. & Leykun B. | April 15, 2024 | Title & abstract |
|  | Prevalence of the major ectoparasites of poultry in extensive and intensive farms in Jimma, Southwestern Ethiopia | Gete B. & Leykun B. | March 19, 2024 | Title & abstract |
|  | Prevalence of Tinea capitis in school going children from Mathare, informal settlement in Nairobi, Kenya | Gete B. & Leykun B. | March 19, 2024 | Title & abstract |
|  | Prevalence of Tunga penetrans infection and other ectoparasites in pigs presented for slaughter at Wambizi Abattoir, Kampala District | Gete B. & Leykun B. | March 19, 2024 | Title & abstract |
|  | Prevalence of tungiasis and its associated factors among primary school children in Karemo division, Siaya county Kenya | Gete B. & Leykun B. | June 20, 2024 | Outcome |
|  | Prevalence of tungiasis and its associated factors among residents of Kipkelion west sub-county; Kericho county, Kenya | Gete B. & Leykun B. | June 20, 2024 | Title & abstract |
|  | Prevalence of Tungiasis and its risk factors of among children of Mettu woreda, southwest Ethiopia, 2020 | Gete B. & Leykun B. | March 19, 2024 | Included |
|  | Prevalence of Tungiasis In Humans In Brazil And In Its Federative Units: A Systematic Review | Gete B. & Leykun B. | March 20, 2024 | Title & abstract |
|  | Prevalence of tungiasis in Oto-Ijanikin village, Badagry, Lagos State, Nigeria | Gete B. & Leykun B. | March 20, 2024 | Title & abstract |
|  | Prevalence of tungiasis in rural poor neighbourhood in Igbokoda, Ondo State, Nigeria | Gete B. & Leykun B. | April 15, 2024 | Included |
|  | Prevalence of tungiasis in the four animal species which were found to be infected with T. penetrans | Gete B. & Leykun B. | March 19, 2024 | Title & abstract |
|  | Prevalence of various dermatoses in school children of Anand district | Gete B. & Leykun B. | March 20, 2024 | Title & abstract |
|  | Prevalence of various skin disorders in school going children of Kashmir valley of North India: a cross-sectional study | Gete B. & Leykun B. | April 15, 2024 | Title & abstract |
|  | Prevalence, intensity and risk factors of tungiasis in Kilifi County, Kenya: I. Results from a community-based study | Gete B. & Leykun B. | March 19, 2024 | Included |
|  | Prevention of tungiasis and tungiasis-associated morbidity using the plant-based repellent Zanzarin: a randomized, controlled field study in rural Madagascar | Gete B. & Leykun B. | July 3, 2024 | Title & abstract |
|  | Prevention of tungiasis using a biological repellent: a small case series | Gete B. & Leykun B. | March 19, 2024 | Title & abstract |
|  | Prophylactic treatment of flea-infested cats with an imidacloprid/flumethrin collar to forestall infection with Dipylidium caninum | Gete B. & Leykun B. | March 19, 2024 | Title & abstract |
|  | Prophylactic treatment of flea-infested dogs with an imidacloprid/flumethrin collar (Seresto®, Bayer) to preempt infection with Dipylidium caninum | Gete B. & Leykun B. | March 15, 2024 | Title & abstract |
|  | Protocol for a Randomized Control Trial for Tungiasis Treatment in Homa Bay County, Kenya: Dimeticone versus Sodium Carbonate. | Gete B. & Leykun B. | June 8, 2024 | Title & abstract |
|  | Pseudoepitheliomatous hyperplasia in ectopic tungiasis: an unusual case | Gete B. & Leykun B. | June 8, 2024 | Title & abstract |
|  | Psychiatric and psychological co-morbidity in patients with dermatologic disorders: epidemiology and management | Gete B. & Leykun B. | June 20, 2024 | Title & abstract |
|  | Psychosocial effect of common skin diseases. | Gete B. & Leykun B. | June 8, 2024 | Title & abstract |
|  | Psycho-social support to children infested by jigger in Kisii County: Social work perspective | Gete B. & Leykun B. | April 15, 2024 | Title & abstract |
|  | Putting the burden of skin diseases on the global map | Gete B. & Leykun B. | April 15, 2024 | Title & abstract |
|  | Qualitative case study of community experiences with Tungiasis in high prevalence villages of Bungoma County, Kenya:“The whole body aches and the jiggers are torturing me!”. | Gete B. & Leykun B. | June 8, 2024 | Title & abstract |
|  | Quality of life issues in children and adolescents with dermatological conditions and their wider impact on the family and society | Gete B. & Leykun B. | June 20, 2024 | Title & abstract |
|  | Quantifying burden of disease caused by tungiasis using disability adjusted life years metric among the children aged 5-14 years in Murang′ a County, Kenya | Gete B. & Leykun B. | June 8, 2024 | Title & abstract |
|  | Recognition and management of common ectoparasitic diseases in travelers | Gete B. & Leykun B. | June 15, 2024 | Title & abstract |
|  | Reconsidering the underestimated burden caused by neglected tropical diseases | Gete B. & Leykun B. | June 15, 2024 | Title & abstract |
|  | Record of Dasypus novemcinctus (Mammalia: Xenarthra) parasited by Tunga terasma (Siphonaptera: Tungidae) in Alegre, State of Espirito Santo, Brazil. | Gete B. & Leykun B. | June 8, 2024 | Title & abstract |
|  | Reduced ectoparasite load, body mass and blood haemolysis in Eurasian kestrels (Falco tinnunculus) along an urban–rural gradient | Gete B. & Leykun B. | June 8, 2024 | Title & abstract |
|  | Reducing prejudice against children with Tungiasis: a qualitative study from Kenya on how a school intervention may raise awareness and change attitudes towards | Gete B. & Leykun B. | June 20 2024 | Title & abstract |
|  | Reflections on Some Human Ectoparasites | Gete B. & Leykun B. | June 20, 2024 | Title & abstract |
|  | Regression of severe tungiasis-associated morbidity after prevention of re-infestation: a case series from rural Madagascar | Gete B. & Leykun B. | June 8, 2024 | Title & abstract |
|  | Relationship between chronic illness on school going children and academic achievement in Ainabkoi County, Kenya | Gete B. & Leykun B. | June 8, 2024 | Title & abstract |
|  | Relationship between jigger infestation and children's participation in school activities in Gatundu district, Kiambu County, Kenya | Gete B. & Leykun B. | June 8, 2024 | Title & abstract |
|  | Repetitive sequences in the ITS1 region of the ribosomal DNA of Tunga penetrans and other flea species (Insecta, Siphonaptera) | Gete B. & Leykun B. | June 8, 2024 | Title & abstract |
|  | Report of a WHO informal meeting on the development of a conceptual framework for tungiasis control: virtual meeting, 11-13 January 2021 | Gete B. & Leykun B. | July 3, 2024 | Title & abstract |
|  | Requirements for the Award of the Degree of Master of Public Health (Monitoring and Evaluation) in the School of Public Health | Gete B. & Leykun B. | June 8, 2024 | Title & abstract |
|  | Research Article Dynamic Behavior of a Stochastic Tungiasis Model for Public Health Education | Gete B. & Leykun B. | April 15, 2024 | Title & abstract |
|  | Residents' corner July 2013. Clues in DeRmosCopy: Entodermoscopy | Gete B. & Leykun B. | June 8, 2024 | Title & abstract |
|  | Resilience as a protective factor for the behavioral problems in school-aged children with atopic dermatitis | Gete B. & Leykun B. | June 8, 2024 | Title & abstract |
|  | Respiratory and dermatological diseases in children with long-term exposure to road traffic immissions | Gete B. & Leykun B. | April 15, 2024 | Title & abstract |
|  | Results of a Double-Blind, Randomized, Placebo-Controlled Phase 1 Study to Evaluate the Safety and Pharmacokinetics of Anti-Zika Virus Immunoglobulin | Gete B. & Leykun B. | March 17, 2024 | Title & abstract |
|  | Revision on tungiasis: treatment options and prevention | Gete B. & Leykun B. | June 20, 2024 | Title & abstract |
|  | Rising Awareness of Tungiasis in Travellers | Gete B. & Leykun B. | July 3, 2024 | Title & abstract |
|  | Risk factors and co-morbidity of skin disorders among female schoolchildren in Eastern Saudi Arabia. | Gete B. & Leykun B. | March 17, 2024 | Title & abstract |
|  | Risk factors associated with Ctenocephalides felis flea infestation of peri-urban goats: a neglected parasite in an under-appreciated host | Gete B. & Leykun B. | April 15, 2024 | Title & abstract |
|  | Risk factors for scabies, tungiasis, and tinea infections among schoolchildren in southern Ethiopia: A cross-sectional Bayesian multilevel model | Gete B. & Leykun B. | March 17, 2024 | Included |
|  | Risk factors for tungiasis in Nigeria: identification of targets for effective intervention | Gete B. & Leykun B. | March 20, 2024 | Title & abstract |
|  | Risk of ectoparasitism and genetic diversity in a wild lesser kestrel population | Gete B. & Leykun B. | March 16, 2024 | Title & abstract |
|  | Robust bi-objective optimal control of tungiasis diseases | Gete B. & Leykun B. | March 17, 2024 | Title & abstract |
|  | Rodent ectoparasites in the Middle East: A systematic review and meta-analysis | Gete B. & Leykun B. | March 16, 2024 | Title & abstract |
|  | Roles and responsibilities veterinary professionals dealing with children in practice. | Gete B. & Leykun B. | March 16, 2024 | Title & abstract |
|  | Sachse MM, Guldbakke KK, Khachemoune A. Tunga penetrans: a stowaway from around the world. | Gete B. & Leykun B. | March 17, 2024 | Title & abstract |
|  | Sand flea (Tunda spp.) infections in humans and domestic animals | Gete B. & Leykun B. | March 16, 2024 | Title & abstract |
|  | Sand flea (Tunga spp.) infections in humans and domestic animals: state of the art | Gete B. & Leykun B. | March 16, 2024 | Title & abstract |
|  | Satellites can reveal global extent of forced labor in the world's fishing fleet | Gete B. & Leykun B. | March 16, 2024 | Title & abstract |
|  | Scabies and pediculosis in Tokelau Island children in New Zealand | Gete B. & Leykun B. | March 17, 2024 | Title & abstract |
|  | Scabies in Koranic schools in Dakar, Senegal: Prevalence and risk factors | Gete B. & Leykun B. | March 8, 2024 | Title & abstract |
|  | Scabies, bedbug, and body lice infestations: a review | Gete B. & Leykun B. | March 8, 2024 | Title & abstract |
|  | Scabies, pediculosis, tungiasis and cutaneous larva migrans in a poor community in northeast Brazil | Gete B. & Leykun B. | March 17, 2024 | Title & abstract |
|  | Schistosomiasis, cercarial dermatitis, and marine dermatitis | Gete B. & Leykun B. | March 17, 202 | Title & abstract |
|  | Scientific literature on neglected tropical diseases: a bibliometric analysis. | Gete B. & Leykun B. | March 17, 2024 | Title & abstract |
|  | Seasonal distribution and common management practices of ectoparasites of domestic dogs in Ilorin, Nigeria | Gete B. & Leykun B. | March 17, 2024 | Title & abstract |
|  | Seasonal variation and persistence of Tungiasis infestation in dogs in an endemic community, Bahia State (Brazil): longitudinal study | Gete B. & Leykun B. | March 17, 2024 | Title & abstract |
|  | Seasonal variation of common skin diseases in pediatric age group a retrospective study conducted in a medical college of Nepal | Gete B. & Leykun B. | March 17, 2024 | Title & abstract |
|  | Seasonal variation of tungiasis in an endemic community | Gete B. & Leykun B. | March 17, 2024 | Title & abstract |
|  | Seasonal variations in the sites of infestation of Chorioptes bovis, a parasitic mite of cattle, with observations on the associated dermatitis | Gete B. & Leykun B. | March 17, 2024 | Title & abstract |
|  | Secondary bacterial infections and antibiotic resistance among tungiasis patients in Western, Kenya | Gete B. & Leykun B. | March 17, 2024 | Title & abstract |
|  | Secondary bacterial infections in patients with atopic dermatitis or other common dermatoses | Gete B. & Leykun B. | March 17, 2024 | Title & abstract |
|  | Selective mass treatment with ivermectin to control intestinal helminthiases and parasitic skin diseases in a severely affected population | Gete B. & Leykun B. | March 17, 2024 | Title & abstract |
|  | Serie parasitosis en Dermatología Tungiasis. | Gete B. & Leykun B. | March 17, 2024 | Title & abstract |
|  | Severe tungiasis in a patient with Klippel-Trenaunay syndrome | Gete B. & Leykun B. | March 17, 2024 | Title & abstract |
|  | Severe tungiasis in underprivileged communities: case series from Brazil. | Gete B. & Leykun B. | March 17, 2024 | Title & abstract |
|  | Severe tungiasis in Western Tanzania: case series | Gete B. & Leykun B. | April 15, 2024 | Title & abstract |
|  | Short Communication: Open Access Ectoparasitic infestations of cats and dogs in Izzi Local Government Area of Ebonyi State, Nigeria: brief communication for ‘One Health’ approach to control of potential zoonoses | Gete B. & Leykun B. | March 17, 2024 | Title & abstract |
|  | Sifonápteros do Brasil, Museu de Zoologia USP/Fapesp, São Paulo | Gete B. & Leykun B. | April 15, 2024 | Title & abstract |
|  | Skin and Hoof lesions on pigs slaughtered and knowledge of abattoir workers on Tungiasis at Wambizzi Co-operative Society Abattoir, Nalukolongo. | Gete B. & Leykun B. | June 8, 2024 | Title & abstract |
|  | Skin conditions among pediatric dermatology outpatients in Botswana | Gete B. & Leykun B. | March 20, 2024 | Title & abstract |
|  | Skin conditions and related need for medical care among persons 1-74 years, United States, 1971-1974 | Gete B. & Leykun B. | June 8, 2024 | Title & abstract |
|  | Skin disease and socioeconomic conditions in rural Africa: Tanzania | Gete B. & Leykun B. | April 20, 2024 | Title & abstract |
|  | Skin Disease Detection for Kids at School Using Deep Learning Techniques. | Gete B. & Leykun B. | April 20, 2024 | Title & abstract |
|  | Skin disease in children: effects on quality of life, stigmatization, bullying, and suicide risk in pediatric acne, atopic dermatitis, and psoriasis patients | Gete B. & Leykun B. | June 8, 2024 | Title & abstract |
|  | Skin disease in the tropics | Gete B. & Leykun B. | March 14, 2024 | Title & abstract |
|  | Skin disease is common in rural Nepal: results of a point prevalence study | Gete B. & Leykun B. | March 15, 2024 | Title & abstract |
|  | Skin disease prevalence study in schoolchildren in rural Côte d'Ivoire: Implications for integration of neglected skin diseases (skin NTDs) | Gete B. & Leykun B. | June 8, 2024 | Title & abstract |
|  | Skin diseases among children attending the outpatient clinic of the University of Nigeria teaching hospital, Enug | Gete B. & Leykun B. | June 8, 2024 | Title & abstract |
|  | Skin diseases among preschool children | Gete B. & Leykun B. | April 20, 2024 | Title & abstract |
|  | Skin diseases and their treatment strategies in sub-saharan african regions | Gete B. & Leykun B. | April 20, 2024 | Title & abstract |
|  | Skin diseases in children in rural Kenya: long‐term results of a dermatology project within the primary health care system | Gete B. & Leykun B. | June 8, 2024 | Title & abstract |
|  | Skin diseases in Greek and immigrant children in Athens | Gete B. & Leykun B. | June 15, 2024 | Title & abstract |
|  | Skin diseases in pediatric patients attending a tertiary dermatology hospital in Northern Tanzania: a cross-sectional study | Gete B. & Leykun B. | June 8, 2024 | Title & abstract |
|  | Skin diseases in returning travelers: etiologies according to clinical presentation. | Gete B. & Leykun B. | June 15, 2024 | Title & abstract |
|  | Skin Disoders of Childhood | Gete B. & Leykun B. | June 15, 2024 | Title & abstract |
|  | Skin disorders among children living in orphanage centres in Dar es Salaam, Tanzania | Gete B. & Leykun B. | June 15, 2024 | Title & abstract |
|  | Skin disorders among male primary school children in Al Hassa, Saudi Arabia: prevalence and socio-demographic correlates-a comparison of urban and rural | Gete B. & Leykun B. | June 8, 2024 | Title & abstract |
|  | Skin disorders among travellers returning from tropical and non-tropical countries consulting a travel medicine clinic | Gete B. & Leykun B. | June 8, 2024 | Title & abstract |
|  | Skin Disorders in Vulnerable Populations | Gete B. & Leykun B. | June 15, 2024 | Title & abstract |
|  | Skin Disorders in Vulnerable Populations: Causes, Impacts and Challenges | Gete B. & Leykun B. | June 15, 2024 | Title & abstract |
|  | Skin infections in returned travelers: an update | Gete B. & Leykun B. | June 15, 2024 | Title & abstract |
|  | Skin lesions in returning travellers | Gete B. & Leykun B. | June 15, 2024 | Title & abstract |
|  | Skin problems in children under five years old at a rural hospital in Southern Ethiopia | Gete B. & Leykun B. | June 15, 2024 | Title & abstract |
|  | Smallholder pig production: prevalence and risk factors of ectoparasites | Gete B. & Leykun B. | June 15, 2024 | Title & abstract |
|  | Social Demographic Factors Influencing the Prevalence of High Tungiasis Infestation among Kilifi Residents, Kenya | Gete B. & Leykun B. | June 10, 2024 | Outcome |
|  | Social research on neglected diseases of poverty: continuing and emerging themes. | Gete B. & Leykun B. |  | Title & abstract |
|  | Sociality, exotic ectoparasites, and fitness in the plural breeding rodent Octodon degus | Gete B. & Leykun B. | June 15, 2024 | Title & abstract |
|  | Socio-demographic characteristics of children infested with scabies in densely populated communities of residential madrashas (Islamic education institutes) in Dhaka | Gete B. & Leykun B. | June 10, 2024 | Title & abstract |
|  | Socio-demographic factors and their association to prevalence of skin diseases among adolescents. | Gete B. & Leykun B. | June 10, 2024 | Title & abstract |
|  | Socio-ecological risk factors associated with human flea infestations of rural household in plague-endemic areas of Madagascar | Gete B. & Leykun B. | June 10, 2024 | Title & abstract |
|  | Socio-Epidemiological Study of Infectious Skin Diseases among School Children in Heet District, Iraq | Gete B. & Leykun B. | June 10, 2024 | Title & abstract |
|  | Soil factors influencing the prevalence of tunga penetrans in Gaichanjiru location of Murang'a county, Kenya | Gete B. & Leykun B. | June 10, 2024 | Outcome |
|  | Some Abiotic Factors Affecting the Survival of the Cat Flea, Ctenocephalides felis (Siphonaptera: Pulicidae) | Gete B. & Leykun B. | April 15, 2024 | Title & abstract |
|  | Some major school health problems in southern Ethiopia: Malnutrition, parasite infections, and skin problems | Gete B. & Leykun B. | June 10, 2024 | Title & abstract |
|  | 'Someone dies in your lap': structural, ecological and political effects on child and maternal health care decisions, Moroto District, Uganda, 2004 | Gete B. & Leykun B. | June 10, 2024 | Title & abstract |
|  | Spatial Distribution of Off-Host Stages of Tunga penetrans in the Soil within the Home Range of Nine Infected Dogs in An Endemic Tourist Area in Brazil. | Gete B. & Leykun B. | June 10, 2024 | Title & abstract |
|  | Spatial distribution, prevalence and potential risk factors of Tungiasis in Vihiga County, Kenya. | Gete B. & Leykun B. | June 10, 2024 | Outcome |
|  | Spectrum of dermatoses among paediatric patients in a teaching hospital of Western Nepal | Gete B. & Leykun B. | June 10, 2024 | Title & abstract |
|  | Spectrum of dermatoses in 165 travelers returning from the tropics with skin diseases | Gete B. & Leykun B. | June 10, 2024 | Title & abstract |
|  | Spectrum of Imported Infectious Diseases: A Comparative Prevalence Study of 16,817 German Travelers and 977 Immigrants from the Tropics and Subtropics. | Gete B. & Leykun B. | June 8, 2024 | Title & abstract |
|  | Spectrum of Skin Diseases among School Aged Children in Jos North-Central Nigeria | Gete B. & Leykun B. | June 8, 2024 | Title & abstract |
|  | Spectrum of skin diseases in Amerindian villages of the Upper Oyapock, French Guiana | Gete B. & Leykun B. | June 8, 2024 | Title & abstract |
|  | Spectrum of skin disorders among primary school children in Umuahia, South-East Nigeria | Gete B. & Leykun B. | June 8, 2024 | Title & abstract |
|  | Stability analysis of the dynamics of tungiasis transmission in endemic areas | Gete B. & Leykun B. | June 8, 2024 | Title & abstract |
|  | Stigma associated with onchocercal skin disease among those affected near the Ofiki and Oyan Rivers in western Nigeria | Gete B. & Leykun B. | June 8, 2024 | Title & abstract |
|  | Stigmatizing Beliefs, Stereotypes and Communication Surrounding Tungiasis in Kenya. | Gete B. & Leykun B. | June 8, 2024 | Title & abstract |
|  | Stowaways with wings: two case reports on high-flying insects | Gete B. & Leykun B. | June 8, 2024 | Title & abstract |
|  | Study on prevalence of ectoparasites of poultry in and around Jimma town | Gete B. & Leykun B. | June 8, 2024 | Title & abstract |
|  | Subungual Hyperpigmented Nodular Lesion in an Adult's Toe | Gete B. & Leykun B. | June 8, 2024 | Title & abstract |
|  | Successful Treatment of Severe Tungiasis in Pigs Using a Topical Aerosol Containing Chlorfenvinphos, Dichlorphos and Gentian Violet. | Gete B. & Leykun B. | June 8, 20224 | Title & abstract |
|  | Sustainable control of tungiasis in rural Nigeria: a case for One Health | Gete B. & Leykun B. | July 2, 2024 | Title & abstract |
|  | The acute vapour inhalation toxicity of 2-butoxyethanol. Points considered when designing and conducting a study in Guinea pigs and evaluating existing inhalation toxicity data on low volatility solvents | Gete B. & Leykun B. | July 2, 2024 | Title & abstract |
|  | The animal reservoir of Tunga penetrans in severely affected communities of north-east Brazil | Gete B. & Leykun B. | July 2, 2024 | Title & abstract |
|  | The assessment of dermatological needs in resource‐poor regions | Gete B. & Leykun B. | April 15, 2024 | Title & abstract |
|  | The biology and ecology of cat fleas and advancements in their pest management: a review | Gete B. & Leykun B. | April 15, 2024 | Title & abstract |
|  | The biology, ecology, and management of the cat flea | Gete B. & Leykun B. | April 15, 2024 | Title & abstract |
|  | The burden of rodent-borne diseases in Africa south of the Sahara | Gete B. & Leykun B. | April 15, 2024 | Title & abstract |
|  | The burden of skin and subcutaneous diseases: findings from the global burden of disease study 2019 | Gete B. & Leykun B. | May 10, 2024 | Title & abstract |
|  | The burden of skin diseases in China: Global Burden of Disease Study 2019 | Gete B. & Leykun B. | March 20, 2024 | Title & abstract |
|  | The Burden of Tungiasis (Jiggers' Infestation) and Its Impact on Rural Vulnerable Populations in Kenya: A Comprehensive Health Assessment on Muranga County | Gete B. & Leykun B. | July 2, 2024 | Title & abstract |
|  | The burden of zoonoses in Paraguay: A systematic review | Gete B. & Leykun B. | July 2, 2024 | Title & abstract |
|  | The causes of skin lesions in the returning travelers: with special reference to Egypt | Gete B. & Leykun B. | July 2, 2024 | Title & abstract |
|  | The Children's Dermatology Life Quality Index (CDLQI): initial validation and practical use | Gete B. & Leykun B. | July 2, 2024 | Title & abstract |
|  | The diagnosis and treatment of tungiasis. | Gete B. & Leykun B. | July 2, 2024 | Title & abstract |
|  | The ectoparasites of some marine birds from Bamfield Marine Station, British Columbia, with particular reference to the common murre, Uria aalge (Pont.) | Gete B. & Leykun B. | March 20, 2024 | Title & abstract |
|  | The effect of allopreening on tick burdens of molting eudyptid penguins | Gete B. & Leykun B. | March 20, 2024 | Title & abstract |
|  | The effect of exogenous testosterone on ectoparasite loads in free‐ranging western fence lizards | Gete B. & Leykun B. | May 10, 2024 | Title & abstract |
|  | The effect of host density on ectoparasite distribution: an example of a rodent parasitized by fleas | Gete B. & Leykun B. | May 10, 2024 | Title & abstract |
|  | The effect of substrate on survival and development of two species of desert fleas (Siphonaptera: Pulicidae) | Gete B. & Leykun B. | May 10, 2024 | Title & abstract |
|  | The effect of the control of endo- and ectoparasites on weight gains in crossbred cattle (Bos taurus taurus × Bos taurus indicus) in the central region of Brazil | Gete B. & Leykun B. | May 10, 2024 | Title & abstract |
|  | The Effectiveness of Topical Dimethicone Together with a One Health Approach for the Control of Tungiasis in the Sanumas Communities, Yanomami Territory, Amazon Rainforest: A Real-World Study | Gete B. & Leykun B. | May 10, 2024 | Title & abstract |
|  | The Effects of Jigger Infestation on School Enrollment and Retention of Children in Rural Areas: A Case of South East Alego Ward, Siaya County Kenya | Gete B. & Leykun B. | March 20, 2024 | Title & abstract |
|  | The Effects of Steinernema carpocapsae (Weiser) Application to Different Life Stages on Adult Emergence of the Cat Flea Ctenocephalides felis (Bouché) | Gete B. & Leykun B. | March 20, 2024 | Title & abstract |
|  | The efficacy of topical, oral and surgical interventions for the treatment of tungiasis: A systematic review of the literature. | Gete B. & Leykun B. | May 10, 2024 | Title & abstract |
|  | The epidemiology of childhood psoriasis: a scoping review | Gete B. & Leykun B. | May 20, 2024 | Title & abstract |
|  | The epidemiology, diagnosis, management, and prevention of ectoparasitic diseases in travelers | Gete B. & Leykun B. | April 20, 2024 | Title & abstract |
|  | The expanded spectrum of bartonellosis in children | Gete B. & Leykun B. | April 20, 2024 | Title & abstract |
|  | The extent of protective footwear use among school-age rural children at high risk for podoconiosis and socio-economic correlates: A household cross-sectional survey in Southern Ethiopia | Gete B. & Leykun B. | May 10, 2024 | Title & abstract |
|  | The fate of the embedded virgin sand flea Tunga penetrans: hypothesis, self-experimentation and photographic sequence. | Gete B. & Leykun B. | April 20, 2024 | Title & abstract |
|  | The fleas (Siphonaptera) in Iran: diversity, host range, and medical importance | Gete B. & Leykun B. | May 10, 2024 | Title & abstract |
|  | The fleas (Siphonaptera) of Fennoscandia and Denmark | Gete B. & Leykun B. | April 10, 2024 | Title & abstract |
|  | The frequency of common skin conditions in preschool-age children in Australia: atopic dermatitis | Gete B. & Leykun B. | May 10, 2024 | Title & abstract |
|  | The genus Tunga Jarocki, 1838 (Siphonaptera: Tungidae). I: taxonomy, phylogeny, ecology and pathogenicity | Gete B. & Leykun B. | March 17, 2024 | Title & abstract |
|  | The global burden of skin disease in 2010: an analysis of the prevalence and impact of skin conditions | Gete B. & Leykun B. | March 20, 2024 | Title & abstract |
|  | The global epidemiology, public health outcomes, management, and prevention of re-emerging ectoparasitic diseases | Gete B. & Leykun B. | March 17, 2024 | Title & abstract |
|  | The impact of COVID-19 pandemic on the prevalence of head lice infestation among children attending schools and kindergartens: direct research in Poland. | Gete B. & Leykun B. | March 15, 2024 | Title & abstract |
|  | The impact of data on effective health policy formulation: a case study of the jigger infestation in Busoga region | Gete B. & Leykun B. | March 17, 2024 | Title & abstract |
|  | The Impact of Neglected Tropical Diseases (NTDs) on Women’s Health and Wellbeing in Sub-Saharan Africa (SSA) | Gete B. & Leykun B. | March 20, 2024 | Title & abstract |
|  | The impact of skin disease on the quality of life of adolescents | Gete B. & Leykun B. | March 20, 2024 | Title & abstract |
|  | The importance of socio-economic status and sex on the prevalence of human pediculosis in government schools children in Lahore, Pakistan | Gete B. & Leykun B. | March 17, 2024 | Title & abstract |
|  | The Influence of Climate Variability on Jigger Infestation in Teso North Sub County, Busia County, Kenya | Gete B. & Leykun B. | March 17, 2024 | Title & abstract |
|  | The infuence of head lice (pediculus humanus capitis) infestation to nutritional status and anemia occurence on female elementary school students | Gete B. & Leykun B. | March 17, 2024 | Title & abstract |
|  | The investigation of foot structure within children who have attention-deficit hyperactivity disorder: a case-controlled study | Gete B. & Leykun B. | April 10, 2024 | Title & abstract |
|  | The Jigger invasion of Busoga Region 2010. | Gete B. & Leykun B. | April 20, 2024 | Title & abstract |
|  | The Maji Maji War and the Prevalence of Diseases in South-Eastern Tanzania, 1905-1910 | Gete B. & Leykun B. | March 20, 2024 | Title & abstract |
|  | The oldest record of flea/armadillos interaction as example of bioerosion on osteoderms from the late Miocene of the Argentine Pampas | Gete B. & Leykun B. | March 17, 2024 | Title & abstract |
|  | The parasitic diseases of school children in Lagos State, Nigeria. | Gete B. & Leykun B. | March 20, 2024 | Title & abstract |
|  | The pattern of dermatological disorders among patients attending the skin OPD of a tertiary care hospital in Kolkata, India | Gete B. & Leykun B. | March 17, 2024 | Title & abstract |
|  | The pattern of skin disorders in a Nigerian tertiary hospital | Gete B. & Leykun B. | April 10, 2024 | Title & abstract |
|  | The patterns of tungiasis in Araruama township, state of Rio de Janeiro, Brazil | Gete B. & Leykun B. | April 20, 2024 | Title & abstract |
|  | The potential risk of HIV infection and transmission of other blood-borne pathogens through the sharing of needles and pins among people infested with jiggers in Kenya | Gete B. & Leykun B. | March 17, 2024 | Title & abstract |
|  | The present status of the conquest of tropical Africa by Pulex irritans Linnaeus, 1758. | Gete B. & Leykun B. | April 10, 2024 | Title & abstract |
|  | The prevalence and association with health-related quality of life of Tungiasis and scabies in schoolchildren in southern Ethiopia | Gete B. & Leykun B. | March 17, 2024 | Included |
|  | The prevalence and descriptive epidemiology of atopic dermatitis in Singapore school children | Gete B. & Leykun B. | April 10, 2024 | Title & abstract |
|  | The Prevalence and Risk Factors Associated with Tungiasis Infestations in Uganda: Implications for Vector Borne and Neglected Tropical Disease Control | Gete B. & Leykun B. | March 17, 2024 | Title & abstract |
|  | The prevalence and risk factors of adolescent acne among schoolchildren in Lithuania: a cross‐sectional study | Gete B. & Leykun B. | March 20, 2024 | Title & abstract |
|  | The prevalence of common skin conditions in Australian school students: 3. acne vulgaris | Gete B. & Leykun B. | March 20, 2024 | Title & abstract |
|  | The prevalence of paediatric skin conditions at a dermatology clinic in KwaZulu-Natal Province over a 3-month period | Gete B. & Leykun B. | March 17, 2024 | Title & abstract |
|  | The prevalence of sand flea (Tunga penetrans) among primary and post-primary school pupils in Choba area of the Niger Delta | Gete B. & Leykun B. | April 16, 2024 | Outcome |
|  | The prevalence of scabies in Monrovia, Liberia: A population-based survey | Gete B. & Leykun B. | March 20, 2024 | Title & abstract |
|  | The prevalence of skin conditions in Romanian school children | Gete B. & Leykun B. | March 10, 2024 | Title & abstract |
|  | The prevalence of skin disease among school children in rural Ethiopia—a preliminary assessment of dermatologic needs | Gete B. & Leykun B. | March 20, 2024 | Title & abstract |
|  | The Prevalence, Risk Factors and Treatment Methods for Tungiasis among Residents of Musokoto Sub-Location, in Kenya | Gete B. & Leykun B. | March 17, 2024 | Included |
|  | The psychosocial impacts of skin-neglected tropical diseases (SNTDs) as perceived by the affected persons: A systematic review | Gete B. & Leykun B. | March 17, 2024 | Title & abstract |
|  | The public health approach to the burden of common skin diseases in the community | Gete B. & Leykun B. | June 10, 2024 | Title & abstract |
|  | The relationship between jiggers infestation and climate variability in teso north, busia county Kenya | Gete B. & Leykun B. | June 5, 2024 | Title & abstract |
|  | The Role of a Social Worker in The Prevention of Jigger Infestation Among Children in Kisii County, Kenya | Gete B. & Leykun B. | June 5, 2024 | Title & abstract |
|  | The role of media in highlighting anti-jigger campaigns in Murang'a South District, Murang'a County | Gete B. & Leykun B. | June 5, 2024 | Title & abstract |
|  | The role of parasitic infections in atopic diseases in rural schoolchildren | Gete B. & Leykun B. | June 5, 2024 | Title & abstract |
|  | The spectrum of dermatological disorders among primary school children in Dar es Salaam | Gete B. & Leykun B. | June 5, 2024 | Title & abstract |
|  | The spectrum of skin diseases in a rural setting in Cameroon (sub-Saharan Africa) | Gete B. & Leykun B. | June 5, 2024 | Title & abstract |
|  | The stigma of skin disease | Gete B. & Leykun B. | June 5, 2024 | Title & abstract |
|  | The striped field mouse (Apodemus agrarius) as a host of fleas (Siphonaptera) and tapeworms (Cestoda) in suburban environment of Lublin (eastern Poland) | Gete B. & Leykun B. | April 20, 2024 | Title & abstract |
|  | The Trick of the Hedgehog: Case Report and Short Review About Archaeopsylla erinacei (Siphonaptera: Pulicidae) in Human Health | Gete B. & Leykun B. | June 5, 2024 | Title & abstract |
|  | The way to cure a complicate syndrome caused by an insect (parasite) using a cerumen extracted from another insect (no parasite, that lives in some countries | Gete B. & Leykun B. | June 20, 2024 | Title & abstract |
|  | Therapeutic Potential of Tea Tree Oil for Tungiasis | Gete B. & Leykun B. | June 20, 2024 | Title & abstract |
|  | Therapy of tungiasis: a double-blinded randomized controlled trial with oral ivermectin | Gete B. & Leykun B. | June 5, 2024 | Title & abstract |
|  | Three-Month-Old Girl With Worm Infestation | Gete B. & Leykun B. | March 10, 2024 | Title & abstract |
|  | Thrombopoietin levels of thrombocytopenic term and preterm newborns with infection | Gete B. & Leykun B. | March 20, 2024 | Title & abstract |
|  | Tick and flea infestations in captive Tapirus terrestris and Tapirus kabomani (Perissodactyla: Tapiridae) in the Brazilian Amazon | Gete B. & Leykun B. | June 5, 2024 | Title & abstract |
|  | Tick-, flea-, and louse-borne diseases of public health and veterinary significance in Nigeria | Gete B. & Leykun B. | April 12, 2024 | Title & abstract |
|  | Time and spatially resolved tracking of the air quality in local public transport | Gete B. & Leykun B. | April 12, 2024 | Title & abstract |
|  | Tinea capitis in schoolchildren in a rural area in southern Ethiopia | Gete B. & Leykun B. | June 5, 2024 | Title & abstract |
|  | Topical ivermectin in the treatment of pediculosis capitis. | Gete B. & Leykun B. | April 12, 2024 | Title & abstract |
|  | Topical treatment of tungiasis: a randomized, controlled trial | Gete B. & Leykun B. | April 12, 2024 | Title & abstract |
|  | Topographic distribution of the sand flea Tunga penetrans in Wistar rats and humans in two endemic areas in Brazil | Gete B. & Leykun B. | June 5, 2024 | Title & abstract |
|  | Toxoplasma gondii infection suppresses house dust mite extract-induced atopic dermatitis in NC/Nga mice | Gete B. & Leykun B. | March 15, 2024 | Title & abstract |
|  | Transmission dynamics of tungiasis in Ethiopia | Gete B. & Leykun B. | March 15, 2024 | Title & abstract |
|  | Transmission networks and ectoparasite mite burdens in Oecomys paricola (Rodentia: Cricetidae) | Gete B. & Leykun B. | June 5, 2024 | Title & abstract |
|  | Travel and migration associated infectious diseases morbidity in Europe, 2008 | Gete B. & Leykun B. | April 20, 2024 | Title & abstract |
|  | Travel- and migration-associated epidermal parasitic skin diseases. A review | Gete B. & Leykun B. | June 5, 2024 | Title & abstract |
|  | Travelers' tropical skin diseases: Challenges and interventions | Gete B. & Leykun B. | April 20, 2024 | Title & abstract |
|  | Treating parasitic skin conditions. | Gete B. & Leykun B. | March 15, 2024 | Title & abstract |
|  | Treatment of animal tungiasis: what's new? | Gete B. & Leykun B. | March 15, 2024 | Title & abstract |
|  | Treatment of human tungiasis with niridazole (Ambilhar) a double-blind placebo-controlled trial | Gete B. & Leykun B. | June 5, 2024 | Title & abstract |
|  | Treatment of parasitic skin diseases with dimeticones a new family of compounds with a purely physical mode of action | Gete B. & Leykun B. | April 15, 2024 | Title & abstract |
|  | Treatment of scabies and pediculosis: facts and controversies | Gete B. & Leykun B. | April 20, 2024 | Title & abstract |
|  | Treatment of tungiasis using a tea tree oil-based gel formulation: protocol for a randomised controlled proof-of-principle trial. | Gete B. & Leykun B. | June 5, 2024 | Title & abstract |
|  | Treatment of tungiasis with a two-component dimeticone: a comparison between moistening the whole foot and directly targeting the embedded sand fleas | Gete B. & Leykun B. | June 5, 2024 | Title & abstract |
|  | Treatment of Tungiasis with dimeticone: a proof-of-principle study in rural Kenya. | Gete B. & Leykun B. | June 24, 2024 | Title & abstract |
|  | Treatment-seeking behaviours of patients with tungiasis in endemic areas of Homa Bay County, Kenya: a mixed-methods study | Gete B. & Leykun B. | June 24, 2024 | Title & abstract |
|  | Tropical dermatology: Venomous arthropods and human skin: Part I. Insecta | Gete B. & Leykun B. | June 28, 2024 | Title & abstract |
|  | Tunga penetrans | Gete B. & Leykun B. | April 16, 2024 | Title & abstract |
|  | Tunga penetrans (Sarcopsylla penetrans) as a cause of agalactia in sows in the Republic of Zaire | Gete B. & Leykun B. | April 16, 2024 | Title & abstract |
|  | Tunga Penetrans (Tungiasis) | Gete B. & Leykun B. | April 16, 2024 | Title & abstract |
|  | Tunga Penetrans A Case Report and Review of the Literature | Gete B. & Leykun B. | June 5, 2024 | Title & abstract |
|  | Tunga penetrans A Silent Setback to Development in Kenya. | Gete B. & Leykun B. | June 20, 2024 | Title & abstract |
|  | Tunga penetrans acquired while traveling in Africa | Gete B. & Leykun B. | June 28, 2024 | Title & abstract |
|  | Tunga penetrans and further parasites in the giant anteater (Myrmecophaga tridactyla) from Minas Gerais, Brazil | Gete B. & Leykun B. | June 20, 2024 | Title & abstract |
|  | Tunga penetrans as a traveler’s disease. | Gete B. & Leykun B. | June 28, 2024 | Title & abstract |
|  | Tunga penetrans causing a rapidly progressing foot ulcer in a patient with uncontrolled type 2 diabetes mellitus | Gete B. & Leykun B. | June 28, 2024 | Title & abstract |
|  | Tunga Penetrans in a Sub-Saharan African Desert Traveler | Gete B. & Leykun B. | April 15, 2024 | Title & abstract |
|  | Tunga penetrans in a young dog imported to Denmark from Brazil; a case report | Gete B. & Leykun B. | April 20, 2024 | Title & abstract |
|  | Tunga Penetrans in Brazil: natural history of Tungiasis in man | Gete B. & Leykun B. | June 28, 2024 | Title & abstract |
|  | Tunga penetrans in Giant anteater (Myrmecophaga tridactyla) from Argentina | Gete B. & Leykun B. | June 28, 2024 | Title & abstract |
|  | Tunga penetrans in south Florida | Gete B. & Leykun B. | March 10, 2024 | Title & abstract |
|  | Tunga penetrans infestation in pigs | Gete B. & Leykun B. | April 15, 2024 | Title & abstract |
|  | Tunga penetrans, Pediculus capitis and Sarcopte scabei infestations among Children< 15yrs in Kamagut Location, Uasin Gishu District, Kenya | Gete B. & Leykun B. | June 28, 2024 | Included |
|  | Tunga penetrans. A case report and review of the literature | Gete B. & Leykun B. | June 20, 2024 | Title & abstract |
|  | Tunga penetrans: a stowaway from around the world. | Gete B. & Leykun B. | June 12, 2024 | Title & abstract |
|  | Tunga penetrans: a tropical surprise in a Dutch toe. | Gete B. & Leykun B. | June 28, 2024 | Title & abstract |
|  | Tunga penetrans: description of a new dermoscopic sign--the radial crown | Gete B. & Leykun B. | May 13, 2024 | Title & abstract |
|  | Tunga penetrans: molecular identification of Wolbachia endobacteria and their recognition by antibodies against proteins of endobacteria from filarial parasites | Gete B. & Leykun B. | June 28, 2024 | Title & abstract |
|  | Tunga penetrans: painful lesions on the feet-the first imported case from Guinea-bissau | Gete B. & Leykun B. | May 13, 2024 | Title & abstract |
|  | Tunga penetrans-A Silent Setback to Development in Kenya. | Gete B. & Leykun B. | May 13, 2024 | Title & abstract |
|  | Tunga Spp. and Tungiasis in Latin America | Gete B. & Leykun B. | May 13, 2024 | Title & abstract |
|  | Tungíase em uma área de aglomerado subnormal de Natal-RN: prevalência e fatores associados. | Gete B. & Leykun B. | June 28, 2024 | Title & abstract |
|  | Tungiasis | Gete B. & Leykun B. | June 28, 2024 | Title & abstract |
|  | Tungiasis - A Janus-faced parasitic skin disease. | Gete B. & Leykun B. | June 28, 2024 | Title & abstract |
|  | Tungiasis (jigger infestation) in rural Kenya, an emerging infectious disease | Gete B. & Leykun B. | June 28, 2024 | Title & abstract |
|  | Tungiasis (jigger infestation) in Rural Kenya, an emerging infectious disease Retro virology | Gete B. & Leykun B. | April 14, 2024 | Title & abstract |
|  | Tungiasis (jigger infestation) in rural Kenya, an emerging infectious disease. | Gete B. & Leykun B. | April 10, 2024 | Title & abstract |
|  | Tungiasis (sand flea disease): a parasitic disease with particular challenges for public health | Gete B. & Leykun B. | June 28, 2024 | Title & abstract |
|  | Tungiasis (Tunga penetrans) in Utah | Gete B. & Leykun B. | June 10, 2024 | Title & abstract |
|  | Tungiasis (tungosis) comes to the Czech Republic | Gete B. & Leykun B. | June 28, 2024 | Title & abstract |
|  | Tungiasis acquired in Chaco Province, Argentina | Gete B. & Leykun B. | April 10, 2024 | Title & abstract |
|  | Tungiasis affects a chilean turist | Gete B. & Leykun B. | April 10, 2024 | Title & abstract |
|  | Tungiasis among children in Kenya is associated with poor nutrition status, absenteeism, poor school performance and high impact on quality of life. | Gete B. & Leykun B. | June 28, 2024 | Outcome |
|  | Tungiasis among five communities in south-western Trinidad, West Indies | Gete B. & Leykun B. | April 16, 2024 | Title & abstract |
|  | Tungiasis among traveler | Gete B. & Leykun B. | April 16, 2024 | Title & abstract |
|  | Tungiasis and cutaneous larva migrans: unpleasant travel souvenirs | Gete B. & Leykun B. | June 28, 2024 | Title & abstract |
|  | Tungiasis and furonculoid myiasis. Jigger and maggot nuisances. | Gete B. & Leykun B. | April 16, 2024 | Title & abstract |
|  | Tungiasis and its Hidden Costs: Investigating the Links to Neurocognitive and Mental Health Outcomes Among Children 8-14 Years of Age in Rural Kenya and Uganda | Gete B. & Leykun B. | March 19, 2024 | Outcome |
|  | Tungiasis and myiasis | Gete B. & Leykun B. | June 28, 2024 | Title & abstract |
|  | Tungiasis and tetanus at the University Hospital Center in Brazzaville. | Gete B. & Leykun B. | March 19, 2024 | Title & abstract |
|  | Tungiasis case after a trip to Kenya | Gete B. & Leykun B. | March 19, 2024 | Title & abstract |
|  | Tungiasis en el área urbana de Popayán, Colombia: reporte de caso | Gete B. & Leykun B. | March 19, 2024 | Title & abstract |
|  | Tungiasis has reached Europe | Gete B. & Leykun B. | April 16, 2024 | Title & abstract |
|  | Tungiasis Has Re-Emerged in Epidemic Dimensions in Recent Years: High Parasite Burden Causing Significant Morbidity | Gete B. & Leykun B. | April 16, 2024 | Title & abstract |
|  | Tungiasis in 5-12 year olds and associated factors in Murang'a South district, Central Kenya | Gete B. & Leykun B. | April 16, 2024 | Title & abstract |
|  | Tungiasis in a 3-year-old child | Gete B. & Leykun B. | April 16, 2024 | Title & abstract |
|  | Tungiasis in a beach volleyball player: a case report | Gete B. & Leykun B. | June 28, 2024 | Title & abstract |
|  | Tungiasis in a Colombian patient | Gete B. & Leykun B. | June 28, 2024 | Title & abstract |
|  | Tungíasis in a dog from a rural community in the state of Maranhão | Gete B. & Leykun B. | June 28, 2024 | Title & abstract |
|  | Tungiasis in a free-ranging jaguar (Panthera onca) population in Brazil. | Gete B. & Leykun B. | April 20, 2024 | Title & abstract |
|  | Tungiasis in a returning traveller from the tropics | Gete B. & Leykun B. | April 20, 2024 | Title & abstract |
|  | Tungiasis in a southern tamandua (Tamandua tetradactyla) from Formosa province, Argentina | Gete B. & Leykun B. | June 28, 2024 | Title & abstract |
|  | Tungiasis in a young child adopted from South America | Gete B. & Leykun B. | June 15, 2024June 15, 2024 | Title & abstract |
|  | Tungiasis in Australia: an exotic disease threat | Gete B. & Leykun B. | June 28, 2024 | Title & abstract |
|  | Tungiasis in Bangou (West Cameroon) | Gete B. & Leykun B. | April 25, 2024 | Title & abstract |
|  | Tungiasis in cañifle residents, suce municipality, sucre state, venezuela | Gete B. & Leykun B. | April 25, 2024 | Title & abstract |
|  | Tungiasis in dogs residing in the Community Nossa Senhora in the Livramento, Sustainable Development Reserve Tupé, Amazonas | Gete B. & Leykun B. | June 28, 2024 | Title & abstract |
|  | Tungiasis in domestic, wild, and synanthropic animals in Brazil | Gete B. & Leykun B. | April 16, 2024 | Title & abstract |
|  | Tungiasis in Haiti: a case series of 383 patients. | Gete B. & Leykun B. | April 16, 2024 | Title & abstract |
|  | Tungiasis in Italy: An imported case of Tunga penetrans and review of the literature. | Gete B. & Leykun B. | April 16, 2024 | Title & abstract |
|  | Tungiasis in Maharashtra (a case report) | Gete B. & Leykun B. | April 16, 2024 | Title & abstract |
|  | Tungiasis in Mendoza | Gete B. & Leykun B. | June 28, 2024 | Title & abstract |
|  | Tungiasis in native Amerindians in Vaupes province: epidemiology, clinical aspects, treatment, and prevention] | Gete B. & Leykun B. | June 28, 2024 | Title & abstract |
|  | Tungiasis in North America | Gete B. & Leykun B. | April 16, 2024 | Title & abstract |
|  | Tungiasis in North America: a report of 2 cases in internationally adopted children | Gete B. & Leykun B. | April 16, 2024 | Title & abstract |
|  | Tungiasis in northern Tanzania: a clinical report from Qameyu village, Babati district, Manyara region | Gete B. & Leykun B. | June 28, 2024 | Title & abstract |
|  | Tungiasis in polyclinical practice in Munich. | Gete B. & Leykun B. | April 16, 2024 | Title & abstract |
|  | Tungiasis in recently arrived African refugees | Gete B. & Leykun B. | April 16, 2024 | Title & abstract |
|  | Tungiasis in rural communities of Badagry Local Government Area, Lagos State, Nigeria | Gete B. & Leykun B. | April 16, 2024 | Title & abstract |
|  | Tungiasis in rural Haiti: a community-based response. | Gete B. & Leykun B. | April 16, 2024 | Title & abstract |
|  | Tungiasis in schoolchildren in Criciuma, Santa Catarina State, South Brazil | Gete B. & Leykun B. | June 28, 2024 | Title & abstract |
|  | Tungiasis in Tennessee | Gete B. & Leykun B. | April 16, 2024 | Title & abstract |
|  | Tungiasis in the past and present: A dire need for intervention | Gete B. & Leykun B. | April 16, 2024 | Title & abstract |
|  | Tungiasis in the Peruvian Aborigines | Gete B. & Leykun B. | June 28, 2024 | Title & abstract |
|  | Tungiasis in the returning traveler | Gete B. & Leykun B. | April 16, 2024 | Title & abstract |
|  | Tungiasis in the Sanumas Amerindians in the Amazon Rainforest, Brazil: Prevalence, Intensity and Morbidity. | Gete B. & Leykun B. | April 16, 2024 | Title & abstract |
|  | Tungiasis in the United States: a travel souvenir | Gete B. & Leykun B. | June 28, 2024 | Title & abstract |
|  | Tungiasis in the urban area of Popayan, Colombia: A case report. | Gete B. & Leykun B. | April 26, 2024 | Title & abstract |
|  | Tungiasis in travelers from tropical Africa | Gete B. & Leykun B. | April 26, 2024 | Title & abstract |
|  | Tungiasis in Trinidad: case report | Gete B. & Leykun B. | April 26, 2024 | Title & abstract |
|  | Tungiasis in two returning travelers in northeastern Brazil: case report | Gete B. & Leykun B. | April 26, 2024 | Title & abstract |
|  | Tungiasis in Western Kenya: Knowledge, Perceptions, and Practices of Household Heads–A Crossectional Study | Gete B. & Leykun B. | April 26, 2024 | Title & abstract |
|  | Tungiasis in Zimbabwe | Gete B. & Leykun B. | April 26, 2024 | Title & abstract |
|  | Tungiasis infection among primary school children in Northeastern Tanzania: prevalence, intensity, clinical aspects and associated factors | Gete B. & Leykun B. | June 28, 2024 | Included |
|  | Tungiasis Infection: Bacterial Secondary Infection and Associated Risk Factors. | Gete B. & Leykun B. | April 26, 2024 | Title & abstract |
|  | Tungiasis infestation in Tanzania | Gete B. & Leykun B. | April 26, 2024 | Title & abstract |
|  | Tungiasis infestation of dermis fat graft in an anophthalmic socket. | Gete B. & Leykun B. | April 26, 2024 | Title & abstract |
|  | Tungiasis Outbreak in Travelers From Madagascar | Gete B. & Leykun B. | April 26, 2024 | Title & abstract |
|  | Tungiasis Outbreak Investigation In Masaiti District, Zambia | Gete B. & Leykun B. | June 28, 2024 | Title & abstract |
|  | Tungiasis presenting as a soft tissue oral lesion | Gete B. & Leykun B. | June 15, 2024 | Title & abstract |
|  | Tungiasis Presenting as Onychomycosis: Probably the First Report of Flea Infestation of the Nail Observed Using Modified Potassium Hydroxide Mount Technique. | Gete B. & Leykun B. | June 15, 2024 | Title & abstract |
|  | Tungiasis presenting with sterile pustular lesions on the hand | Gete B. & Leykun B. | June 28, 2024 | Title & abstract |
|  | Tungiasis presenting with whitish nodules on the feet | Gete B. & Leykun B. | June 15, 2024 | Title & abstract |
|  | Tungiasis Risk Factors in Rural Community in Murang'a County, Kenya | Gete B. & Leykun B. | June 15, 2024 | Title & abstract |
|  | Tungiasis Stigma and Control Practices in a Hyperendemic Region in Northeastern Uganda. | Gete B. & Leykun B. | June 15, 2024 | Title & abstract |
|  | Tungiasis under dermoscopy: in vivo and ex vivo examination of the cutaneous infestation due to Tunga penetrans | Gete B. & Leykun B. | June 28, 2024 | Title & abstract |
|  | Tungiasis-" Vacation dermatitis" | Gete B. & Leykun B. | June 15, 2024 | Title & abstract |
|  | Tungiasis, a Nuisance to the Marginalized Population of Developing Countries | Gete B. & Leykun B. | June 15, 2024 | Title & abstract |
|  | Tungiasis, a rare case of plantar inflammatory disease, a review of travelers skin lesions for emergency providers | Gete B. & Leykun B. | June 28, 2024 | Title & abstract |
|  | Tungiasis, an emerging tropical disease in Chile: three imported cases reports | Gete B. & Leykun B. | June 15, 2024 | Title & abstract |
|  | Tungiasis, an uncommon ectoparisitic disease | Gete B. & Leykun B. | June 15, 2024 | Title & abstract |
|  | Tungiasis, una enfermedad tropical emergente en Chile: Comunicación de tres casos clínicos importados | Gete B. & Leykun B. | June 28, 2024 | Title & abstract |
|  | Tungiasis. A Case of Diagnosis of Tropical Dermatosis | Gete B. & Leykun B. | April 20, 2024 | Title & abstract |
|  | Tungiasis. A clinical case | Gete B. & Leykun B. | April 20, 2024 | Title & abstract |
|  | Tungiasis. Considerations on a case with intense infestation. | Gete B. & Leykun B. | June 28, 2024 | Title & abstract |
|  | Tungiasis. Presentación de un caso clínico [Tungiasis: a case report] | Gete B. & Leykun B. | May 24, 2024 | Title & abstract |
|  | Tungiasis: "the greatest curse that has ever afflicted Africa" | Gete B. & Leykun B. | June 28, 2024 | Title & abstract |
|  | Tungiasis: a case report | Gete B. & Leykun B. | April 20, 2024 | Title & abstract |
|  | Tungiasis: a highly neglected disease among neglected diseases. Case series from Nduta refugee camp (Tanzania) | Gete B. & Leykun B. | May 24, 2024 | Title & abstract |
|  | Tungiasis: a neglected disease causing severe morbidity in a shantytown in Fortaleza, State of Ceara] | Gete B. & Leykun B. | April 20, 2024 | Title & abstract |
|  | Tungiasis: A Neglected Disease Related to Poverty | Gete B. & Leykun B. | May 24, 2024 | Title & abstract |
|  | Tungiasis: a neglected epidermal parasitic skin disease of marginalized populations--a call for global science and policy | Gete B. & Leykun B. | May 24, 2024 | Title & abstract |
|  | Tungiasis: a neglected health problem in rural Cameroon | Gete B. & Leykun B. | April 20, 2024 | Title & abstract |
|  | Tungiasis: a neglected health problem of poor communities | Gete B. & Leykun B. | May 24, 2024 | Title & abstract |
|  | Tungiasis: a poorly documented tropical dermatosis | Gete B. & Leykun B. | May 24, 2024 | Title & abstract |
|  | Tungiasis: a poorly-known diagnosis in Europe. Two paradigmatic cases from Portugal | Gete B. & Leykun B. | June 10, 2024 | Title & abstract |
|  | Tungiasis: a rare consequence of the fascination by the exotic? | Gete B. & Leykun B. | May 24, 2024 | Title & abstract |
|  | Tungiasis: A Rare Parasitic Infestation in Genitals of a Native Male from Kathmandu | Gete B. & Leykun B. | May 24, 2024 | Title & abstract |
|  | Tungiasis: a report of four new cases and a review of imported cases in Italy in the last thirty years | Gete B. & Leykun B. | May 24, 2024 | Title & abstract |
|  | Tungiasis: an epidermal parasitic disease of the skin. brief relate | Gete B. & Leykun B. | June 20, 2024 | Title & abstract |
|  | Tungiasis: an infrequent consequence of overseas travel? | Gete B. & Leykun B. | June 20, 2024 | Title & abstract |
|  | Tungiasis: an overview | Gete B. & Leykun B. | May 24, 2024 | Title & abstract |
|  | Tungiasis: An Underdiagnosed Problem | Gete B. & Leykun B. | June 20, 2024 | Title & abstract |
|  | Tungiasis: Another Traveler's Concern | Gete B. & Leykun B. | June 20, 2024 | Title & abstract |
|  | Tungiasis: Biology, Life Cycle, Epidemiology, Diagnosis, Prevention, and Treatment | Gete B. & Leykun B. | May 24, 2024 | Title & abstract |
|  | Tungiasis: Case Report of a Traveller to Kenya | Gete B. & Leykun B. | June 20, 2024 | Title & abstract |
|  | Tungiasis: Clinical, Dermoscopic and Histopathological Findings in 13 Cases from Communities of Extreme Poverty in Paraguay | Gete B. & Leykun B. | June 20, 2024 | Title & abstract |
|  | Tungiasis: consequences of delayed presentation/diagnosis | Gete B. & Leykun B. | July 1, 2024 | Title & abstract |
|  | Tungiasis: diagnosis at a glance. | Gete B. & Leykun B. | March 15, 2024 | Title & abstract |
|  | Tungiasis: ectoparasitosis importada | Gete B. & Leykun B. | July 1, 2024 | Title & abstract |
|  | Tungiasis: eggs seen with dermoscopy | Gete B. & Leykun B. | March 15, 2024 | Title & abstract |
|  | Tungiasis: high prevalence, parasite load, and morbidity in a rural community in Lagos State, Nigeria | Gete B. & Leykun B. | July 1, 2024 | Included |
|  | Tungiasis: imported disease | Gete B. & Leykun B. | March 15, 2024 | Title & abstract |
|  | Tungiasis: more than an exotic nuisance | Gete B. & Leykun B. | July 1, 2024 | Title & abstract |
|  | Tungiasis: Neglected Diseases of Resource-Poor Community | Gete B. & Leykun B. | March 15, 2024 | Title & abstract |
|  | Tungiasis: Outbreak investigation of a zoonosis during overseas deployment | Gete B. & Leykun B. | July 1, 2024 | Title & abstract |
|  | Tungiasis: Participation of Cats and Chickens in the Dispersion and Maintenance of the Disease in an Endemic Tourist Area in Brazil | Gete B. & Leykun B. | July 1, 2024 | Title & abstract |
|  | Tungiasis: report of one case and review of the 14 reported cases in the United States | Gete B. & Leykun B. | March 15, 2024 | Title & abstract |
|  | Tungiasis: tratamiento de un caso con invermectina | Gete B. & Leykun B. | March 16, 2024 | Title & abstract |
|  | Tungiasis: una rara ectoparasitosis. Caso clínico | Gete B. & Leykun B. | July 1, 2024 | Title & abstract |
|  | Tungiasis: unarara ectoparasitosis. Caso clínico [Tungiasis | Gete B. & Leykun B. | March 16, 2024 | Title & abstract |
|  | Tungiasis:“the greatest curse that has ever afflicted Africa” | Gete B. & Leykun B. | July 1, 2024 | Title & abstract |
|  | Tungiasis--a cause of painful feet in a tropical traveller | Gete B. & Leykun B. | March 16, 2024 | Title & abstract |
|  | Tungiasis–a Janus-faced parasitic skin disease | Gete B. & Leykun B. | March 16, 2024 | Title & abstract |
|  | Tungiasis–a Janus-faced parasitic skin disease | Gete B. & Leykun B. | July 1, 2024 | Title & abstract |
|  | Tungiasis--a middle European rare skin parasitosis. A case report. | Gete B. & Leykun B. | March 16, 2024 | Title & abstract |
|  | Tungiasis--a neglected disease with many challenges for global public health. P | Gete B. & Leykun B. | July 1, 2024 | Title & abstract |
|  | Tungiasis-A threat to the travelers feet | Gete B. & Leykun B. | June 10, 2024 | Title & abstract |
|  | Tungiasis--an increasingly frequent vacation dermatosis. | Gete B. & Leykun B. | July 1, 2024 | Title & abstract |
|  | Tungiasis--an unusual case of severe infestation | Gete B. & Leykun B. | March 16, 2024 | Title & abstract |
|  | Tungiasis-associated morbidity in pigs and dogs in endemic villages of Uganda | Gete B. & Leykun B. | June 10, 2024 | Title & abstract |
|  | Tungiasis-related knowledge and treatment practices in two endemic communities in northeast Brazil | Gete B. & Leykun B. | July 1, 2024 | Title & abstract |
|  | Tungiasis-related life quality impairment in children living in rural Kenya | Gete B. & Leykun B. | June 10, 2024 | Title & abstract |
|  | Tungiasis-the first case of imported arthropodic infection diagnosed in Lithuania. | Gete B. & Leykun B. | June 10, 2024 | Title & abstract |
|  | Tungiasis-Traveler's ectoparasitosis of the foot: A case report | Gete B. & Leykun B. | July 1, 2024 | Title & abstract |
|  | Tungiosis adquirida en la pro- vincia del Chaco, Argentina [Tungiasis acquired in Chaco Province, Argentina. | Gete B. & Leykun B. | July 1, 2024 | Title & abstract |
|  | Two cases of imported tungiasis with severe Staphylococcus aureus superinfection | Gete B. & Leykun B. | June 10, 2024 | Title & abstract |
|  | Two contagious ectoparasites in an orphanage children in Nasr City, Cairo. | Gete B. & Leykun B. | June 10, 2024 | Title & abstract |
|  | Two Severe Cases of Tungiasis in Goat Kids in Uganda | Gete B. & Leykun B. | June 10, 2024 | Title & abstract |
|  | Typical histologic features of Tunga penetrans in skin biopsies. | Gete B. & Leykun B. | April 14, 2024 | Title & abstract |
|  | Ubiquitin proteasomal system is a potential target of the toxic effects of organophosphorus flame retardant triphenyl phosphate | Gete B. & Leykun B. | March 16, 2024 | Title & abstract |
|  | Ultrastructural findings in tungiasis | Gete B. & Leykun B. | April 14, 2024 | Title & abstract |
|  | Uncommon human urinary tract myiasis due to Psychoda sp. Larvae, Kashan, Iran: A case report | Gete B. & Leykun B. | May 16, 2024 | Title & abstract |
|  | Understanding foot conditions, morphologies and functions in children: a current review | Gete B. & Leykun B. | May 16, 2024 | Title & abstract |
|  | University social projection as an axis for the prevention of parasitic infestations in marginal communities of Peru. | Gete B. & Leykun B. | April 14, 2024 | Title & abstract |
|  | Unusual cutaneous infectious and parasitic diseases | Gete B. & Leykun B. | May 16, 2024 | Title & abstract |
|  | Unusual dermal arthropod infestations | Gete B. & Leykun B. | May 16, 2024 | Title & abstract |
|  | Update of knowledge on Neglected Diseases in Haiti: Mansonelliasis, Tungiasis, Leprosy, and Anthrax | Gete B. & Leykun B. | April 14, 2024 | Title & abstract |
|  | Update on parasitic dermatoses. | Gete B. & Leykun B. | March 25, 2024 | Title & abstract |
|  | Updates on Scabies and Pediculosis with Emphasis on Pediatric Age Group | Gete B. & Leykun B. | May 16, 2024 | Title & abstract |
|  | Urban stray cats infested by ectoparasites with zoonotic potential in Greece | Gete B. & Leykun B. | March 25, 2024 | Title & abstract |
|  | Use of Botulism Antitoxin Heptavalent (A, B, C, D, E, F, G)-(Equine) (BAT(®)) in Clinical Study Subjects and Patients: A 15-Year Systematic Safety Review | Gete B. & Leykun B. | April 14, 2024 | Title & abstract |
|  | Use of sarolaner in the treatment of tungiasis in naturally infested dogs | Gete B. & Leykun B. | May 16, 2024 | Title & abstract |
|  | Use of the WHO Access, Watch, and Reserve classification to define patterns of hospital antibiotic use (AWaRe): an analysis of paediatric survey data from 56 countries | Gete B. & Leykun B. | March 25, 2024 | Title & abstract |
|  | Vacation dermatoses | Gete B. & Leykun B. | April 14, 2024 | Title & abstract |
|  | Vaccuuming method as a successful strategy in the diagnosis of active infestation by Pediculus humanus capitis | Gete B. & Leykun B. | May 26, 2024 | Title & abstract |
|  | Variability of dermoscopic features of tungiasis | Gete B. & Leykun B. | April 14, 2024 | Title & abstract |
|  | Vector control has a role to play in mitigating the high incidence of flea-borne typhus in Los Angeles County, California | Gete B. & Leykun B. | March 25, 2024 | Title & abstract |
|  | Vector-and rodent-borne diseases in Europe and North America: Distribution, public health burden, and control | Gete B. & Leykun B. | April 14, 2024 | Title & abstract |
|  | Vector-borne diseases in South-East Asia: burdens and key challenges to be addressed | Gete B. & Leykun B. | March 25, 2024 | Title & abstract |
|  | Vector-borne parasitic zoonoses: emerging scenarios and new perspectives | Gete B. & Leykun B. | March 25, 2024 | Title & abstract |
|  | Vegetable Butters and Oils as Therapeutically and Cosmetically Active Ingredients for Dermal Use: A Review of Clinical Studies. | Gete B. & Leykun B. | April 14, 2024 | Title & abstract |
|  | Verrucous tungiasis - image and clinical findings in a patient with an exuberant case | Gete B. & Leykun B. | May 26, 2024 | Title & abstract |
|  | Very severe tungiasis in Amerindians in the Amazon lowland of Colombia: A case series. | Gete B. & Leykun B. | May 26, 2024 | Title & abstract |
|  | We Need to Go Back to Our Schools, and We Need to Make that Change We Wish to See’: Empowering Teachers for Disability Inclusion. | Gete B. & Leykun B. | March 25, 2024 | Title & abstract |
|  | Web GIS as a DIsease Management Workspace: Enabling Advocacy at Multiple Scales Across Multiple Continents with the Case of Tungiasis | Gete B. & Leykun B. | May 26, 2024 | Title & abstract |
|  | What's new in travel-associated dermatology? | Gete B. & Leykun B. | March 10, 2024 | Title & abstract |
|  | When is a parasite not a parasite? Effects of larval tick burdens on white‐footed mouse survival | Gete B. & Leykun B. | March 10, 2024 | Title & abstract |
|  | 'Whitish chains': a remarkable in vivo dermoscopic finding of tungiasis | Gete B. & Leykun B. | May 26, 2024 | Title & abstract |
|  | Willingness to pay for footwear, and associated factors related to podoconiosis in northern Ethiopia. | Gete B. & Leykun B. | March 10, 2024 | Title & abstract |
|  | Wolbachia infection in the newly described Ecuadorian sand flea, Tunga trimamillata | Gete B. & Leykun B. | July 3, 2024 | Title & abstract |
|  | World Association for the Advancement of Veterinary Parasitology (WAAVP): Guideline for evaluating the efficacy of parasiticides against ectoparasites | Gete B. & Leykun B. | June 8, 2024 | Title & abstract |
|  | World Health Organization strategic framework for integrated control and management of skin-related neglected tropical diseases: what does this mean for dermatologists? | Gete B. & Leykun B. | March 10, 2024 | Title & abstract |
|  | Years lost due to disability from skin diseases in China 1990–2017: Findings from the Global Burden of Disease Study 2017 | Gete B. & Leykun B. | May 23, 2024 | Title & abstract |
|  | Young Woman With Black Spot on Foot | Gete B. & Leykun B. | March 10, 2024 | Title & abstract |
