# Supplementary material for: Prevalence and factors associated with Tungiasis among school age children in Sub Saharan Africa: A systematic review and meta-analysis
Source: PLoS One. 2025 May 8;20(5):e0321513. doi: 10.1371/journal.pone.0321513 (PMC12061120; doi:10.1371/journal.pone.0321513)
Supplement: Attachment — Submitted filename: Rebuttal letter.docx [file pone.0321513.s006.docx]

Rebuttal letter

**Response to journal requirement**

**#1.** Please ensure that your manuscript meets PLOS ONE's style requirements, including those for file naming.

**Response: thank you very much for your concern. Hence, we have tried to revise the manuscript based on the requirement of the journal.**

**#2.** We note that your Data Availability Statement is currently as follows: [All relevant data are within the manuscript and its Supporting Information files.] Please confirm at this time whether or not your submission contains all raw data required to replicate the results of your study. Authors must share the “minimal data set” for their submission.

**Response: thank you very much for the confusion we have created. Therefore, we have modified the data availability statement based on the requirement of the PLOS ONE.**

**#3.** As required by our policy on Data Availability, please ensure your manuscript or supplementary information includes the following: A numbered table of all studies identified in the literature search, including those that were excluded from the analyses.

**Response: Thank you very much for we have incorporated all the data required for the clarity of the data extraction process.**

**#4.** Please review your reference list to ensure that it is complete and correct. If you have cited papers that have been retracted, please include the rationale for doing so in the manuscript text, or remove these references and replace them with relevant current references. Any changes to the reference list should be mentioned in the rebuttal letter that accompanies your revised manuscript. If you need to cite a retracted article, indicate the article’s retracted status in the References list and also include a citation and full reference for the retraction notice.

**Response: We have reviewed all the references and no reference is retracted. Therefore, we have used all the references as it stands and no references are retracted.**

**Response to reviewer one**

#1: The author has done so well in terms of literature review but there is a problem in presenting the information. For example, there is s mix up in the literature right from the introduction. The introduction should talk about the prevalence of Tungiasis and its risk factors. Later it should at least explain what the gap is and how the study is going to deal with the gap
On the methodology, there is repetition on the study setting, the information search is a good one but should be shortened, there is a mix up on the eligibility criteria and main methodology. It would be good if the author uses a diagram in describing the searching process. A good table would also do in explaining the sub group analysis. There are some grammatical errors in the results and also in the discussion.

**Response: thank you very much for your constructive comment. We have includes all the comments and concerned raised by the reviewer. See the revised version of the manuscript. Since the aim of this systematic review and meta-analysis, focuses both on prevalence’s and factors associated with the outcome variable of our interest, the introduction section includes all these issues under consideration.**
